# MpsAB is important for *Staphylococcus aureus* virulence and growth at atmospheric CO_2_ levels

**DOI:** 10.1038/s41467-019-11547-5

**Published:** 2019-08-09

**Authors:** Sook-Ha Fan, Patrick Ebner, Sebastian Reichert, Tobias Hertlein, Susanne Zabel, Aditya Kumar Lankapalli, Kay Nieselt, Knut Ohlsen, Friedrich Götz

**Affiliations:** 1 Microbial Genetics, Interfaculty Institute of Microbiology and Infection Medicine Tübingen (IMIT), University of Tübingen, Auf der Morgenstelle 28, D-72076 Tübingen, Germany; 2 Institute for Molecular Infection Biology (IMIB), University of Würzburg, Josef-Schneider-Strasse 2, D-97080 Würzburg, Germany; 3 Center for Bioinformatics Tübingen, University of Tübingen, Sand 14, D-72076 Tübingen, Germany; 4 Department of Archaeogenetics, Max Planck Institute for the Science of Human History, Kahlaische Strasse 10, D-07745 Jena, Germany

**Keywords:** Bacterial physiology, Bacteriology, Pathogens

## Abstract

The mechanisms behind carbon dioxide (CO_2_) dependency in non-autotrophic bacterial isolates are unclear. Here we show that the *Staphylococcus aureus mpsAB* operon, known to play a role in membrane potential generation, is crucial for growth at atmospheric CO_2_ levels. The genes *mpsAB* can complement an *Escherichia coli* carbonic anhydrase (CA) mutant, and CA from *E. coli* can complement the *S. aureus* delta-*mpsABC* mutant. In comparison with the wild type, *S. aureus mps* mutants produce less hemolytic toxin and are less virulent in animal models of infection. Homologs of *mpsA* and *mpsB* are widespread among bacteria and are often found adjacent to each other on the genome. We propose that MpsAB represents a dissolved inorganic carbon transporter, or bicarbonate concentrating system, possibly acting as a sodium bicarbonate cotransporter.

## Abstract (web summary)

The mechanisms behind CO_2_ dependency in non-autotrophic bacterial isolates are unclear. Here the authors show that the *Staphylococcus aureus mpsAB* operon is crucial for growth at atmospheric CO_2_ levels, and provide evidence indicating that MpsAB represents a bicarbonate concentrating system.

## Introduction

Early concepts of the function of CO_2_ in bacterial growth can be traced back to as early as a century ago. However, convincing experimental evidence only surfaced in 1935 when Gladstone reported that CO_2_ is an essential factor in the growth of bacteria^1^. The growth-promoting effect of CO_2_ was observed in various bacteria, including *Staphylococcus aureus*, particularly when glucose was substituted by another carbon source. Many years later, CO_2_-dependent *S. aureus* was isolated from abscesses and osteomyelitis^2,3^. These strains, though very rare, were well documented as dwarf or gonidial (G) strains and usually produced tiny colonies in the absence of additional CO_2_^4^. Although G strains can be isolated from lesions, their virulence is low in a mouse infection model^3^. Since the early 1970’s, the CO_2_ dependence of staphylococci has been largely overlooked due in part to the routine cultivation of clinical isolates in CO_2_ incubators.

Renewed interest in these so-called dwarf colonies, now referred to as small colony variants (SCVs), was initiated in large part because they were frequently associated with persistent and relapsing infections such as airway infections and osteomyelitis^5–7^. In most cases, SCVs consist of mutants that are frequently affected in respiration comprising thymidine, hemin, or menadione auxotrophs^8–10^. However, unlike the earlier dwarf mutants described by Slifkin^3^ the respiratory mutants cannot be complemented by elevated CO_2_. There are several reports where CO_2_-dependent SCVs of *S. aureus* have been isolated from patients with catheter-related bacteremia, endocarditis, wound infections, respiratory infections, or nasal colonization^11^. This shows that CO_2_-dependent SCVs play a role in certain infections and further studies are necessary to unravel the genetic and biochemical basis of this type of auxotrophy.

We previously investigated whether *S. aureus* possesses a type 1 electrogenic NADH:quinone oxidoreductase (Ndh1), a proton pump that translocates cations, such as H^+^ or Na^+^ ^12^. It turned out that such an Ndh1 is not present in staphylococci; however, we found *S. aureus* has a homolog of the *Escherichia coli* specific NuoL protein, one of the core components of the proton pumping mechanism in *E. coli*^13^. We named the gene *mpsA* (membrane potential-generating system), because an *mpsA* mutant was severely defected in membrane potential generation and also grew like an SCV^12^. This connection and in light of the recent attention on SCVs, we sought to determine the function of the *mps* operon.

In *S. aureus, mpsA* is cotranscribed with the *mpsB* gene, and is separated by a weak transcription terminator from the downstream *mpsC*. It was unknown at that time whether *mpsC* contributes to the MpsAB function. MpsA, like the NuoL homolog is a membrane protein with predicted 14 transmembrane domains, while MpsB is a cytoplasmic protein with a predicted metal binding site. While MpsABC lacks NADH oxidation activity, it participates in Na^+^ transport as *mpsABC* complemented an Na^+^/H^+^ antiporter-deficient *E. coli* strain, suggesting that MpsABC constitutes a cation-translocating system^12^.

Here, we extend our previous observations by analyzing the function of MpsABC in greater detail. In principle, this study builds on the work of the 1970s, in which dwarf colonies were found to be complemented by CO_2_. We show that MpsAB increase the uptake of bicarbonate and represents a new class of dissolved inorganic carbon (DIC) transporter. DIC transporters are responsible for creating an elevated concentration of intracellular bicarbonate and supplying it to carboxysomes and Rubisco. Such transporters are most well studied in cyanobacteria^14^, but are not fully described in other autotrophic bacteria. In *S. aureus*, SCV-like *mpsAB* mutants cannot supply and store sufficient bicarbonate from atmospheric air to feed the important carboxylase reactions in central metabolism. The severe growth defect can only be compensated by increased CO_2_/bicarbonate supplementation. Importantly, MpsAB homologs are widespread in bacteria and were recently found in autotrophic bacteria functioning as DIC transporters to supply carboxysomes and RubisCO with bicarbonate/CO_2_^15,16^.

## Results

### Growth defect of Δ*mpsABC* is rescued by elevated CO_2_/NaHCO_3_

**Fig. 1:**
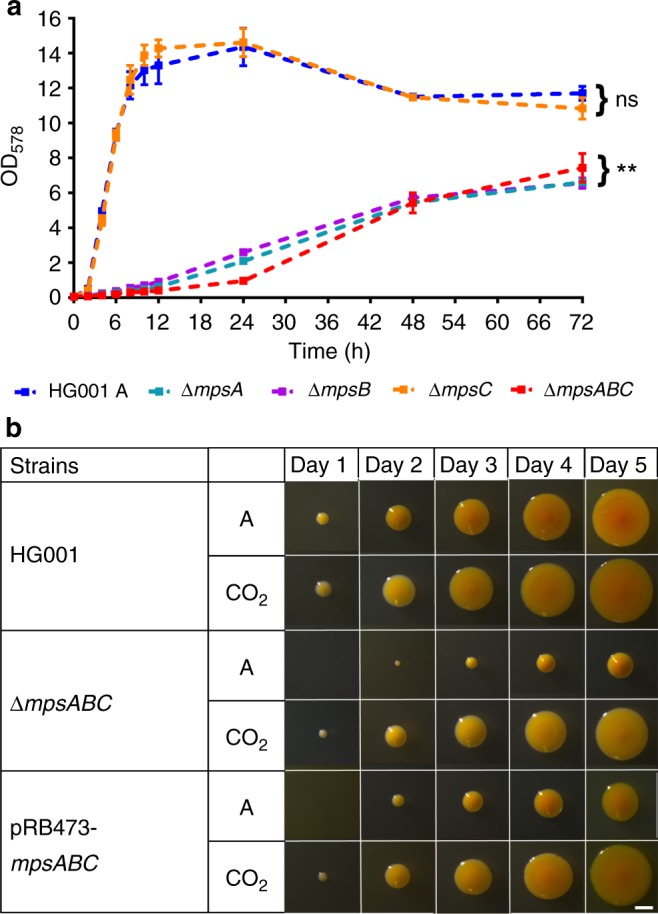
Growth of HG001 *∆mpsABC*. **a** Growth of wild type HG001, *∆mpsA, ∆mpsB, ∆mpsC*, and *∆mpsABC* in atmospheric (A) conditions. The growth of all mutants except *∆mpsC* was significantly lower compared to HG001, **p < 0.01 by unpaired two-sided *t*-test. *∆mpsC* showed no significant difference (ns) in growth compared to HG001. Each point shown in the graph represents the mean value ± standard error mean (SEM) from three independent experiments. Source data are provided as a Source Data file. **b** Colony appearances of wild type HG001, *∆mpsABC*, and its complemented mutant (plasmid pRB473-*mpsABC*) during 5 days of incubation in atmospheric (A) conditions and 5% CO_2_. White bar represents a scale of 1 mm

As previously reported, deletion of the *mpsABC* operon in *S. aureus* strain SA113 resulted in a severe growth defect, with the typical SCV-like phenotype^12^. To further characterize this operon, we constructed single deletion mutants of Δ*mpsA*, Δ*mpsB*, and Δ*mps*C, as well as a deletion of the entire operon, Δ*mpsABC*, in the *agr*-positive *S. aureus* strain HG001^17^. The mutants were analyzed for growth in liquid culture under atmospheric growth conditions. As shown in Fig. 1a, the mutants Δ*mpsA*, Δ*mpsB*, and Δ*mpsABC* grew slowly and did not achieve the final OD of the wild type (WT) strain HG001, even after 72 h. The growth delay was most pronounced in the first 24 h. In contrast to Δ*mpsA* and Δ*mpsB*, the growth of Δ*mpsC* was not affected, indicating that *mpsC* does not contribute to the *mpsAB* activity.

**Fig. 2:**
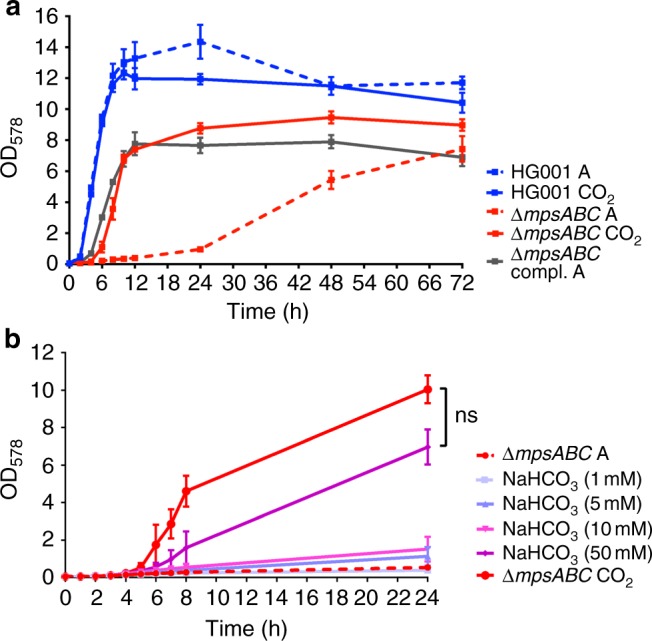
Growth of HG001 *∆mpsABC* can be complemented with 5% CO_2_ and NaHCO_3_. **a** Growth of HG001, *∆mpsABC* in atmospheric (A)conditions and 5% CO_2_. The growth of the mutant can also be complemented by plasmid pRB473 carrying *mpsABC* (compl.). Each point shown in the graph represents the mean value ± standard error mean (SEM) from three independent experiments. **b** Growth of *∆mpsABC* in atmospheric (A) and with additions of NaHCO_3_ ranging from concentrations of 1 to 50 mM, and 5% CO_2_. The growth of *∆mpsABC* with addition of 50 mM NaHCO_3_ was similar to that under 5% CO_2_, as there was no significance difference (ns) between the two conditions (*p* > 0.05) by unpaired two-sided *t*-test. Each point shown in the graph represents the mean value ± standard error mean (SEM) from two independent experiments. Source data are provided as a Source Data file

Interestingly, growth of all mutants could be restored to near WT levels under elevated (5%) CO_2_ concentrations. During the course of 5 days incubation in 5% CO_2_, the colonies of all mutants were significantly larger than those grown in atmospheric air. Growth of the WT strain was largely uninfluenced by CO_2_, although the colony size was slightly increased (Fig. 1b and Supplementary Fig. 1). The growth-promoting effect of CO_2_ was also seen in liquid medium (Fig. 2a and Supplementary Fig. 2). Comparison of the growth in tryptic soy broth (TSB) in the absence or presence of 5% CO_2_ revealed that growth of the *∆mpsABC* mutant was significantly improved in the presence of CO_2_ (Fig. 2a). This growth tendency is similar in the complemented mutant *∆mpsABC*(pRB473-*mpsABC*) under atmospheric conditions. Given that CO_2_ and bicarbonate are interconvertible in aqueous solution, we also attempted to complement *∆mpsABC* with sodium bicarbonate (NaHCO_3_). NaHCO_3_ at concentration ranging from 1 to 50 mM were added to the growth medium and growth of *∆mpsABC* was followed for 24 h. At low concentrations (1 mM NaHCO_3_) no difference in the growth of *∆mpsABC* was observed (Fig. 2b). However, with increasing concentrations of up to 50 mM NaHCO_3_, its growth increased steadily before reaching final OD values comparable with those of 5% CO_2_. Similar observation was also seen with *∆mpsB* (Supplementary Fig. 3a and 3b). The pH of the medium (TSB) increased with the addition of NaHCO_3_, however the effect of pH of the medium did not play a crucial role on the growth of Δ*mpsABC* (see Supplementary Note 1, Supplementary Table 3 and Supplementary Fig. 8). The results indicate that the growth deficiency of *∆mpsABC* can be largely compensated by the addition of 5% CO_2_ and high concentrations of NaHCO_3_.

### MpsABC increase the uptake of bicarbonate

**Fig. 3:**
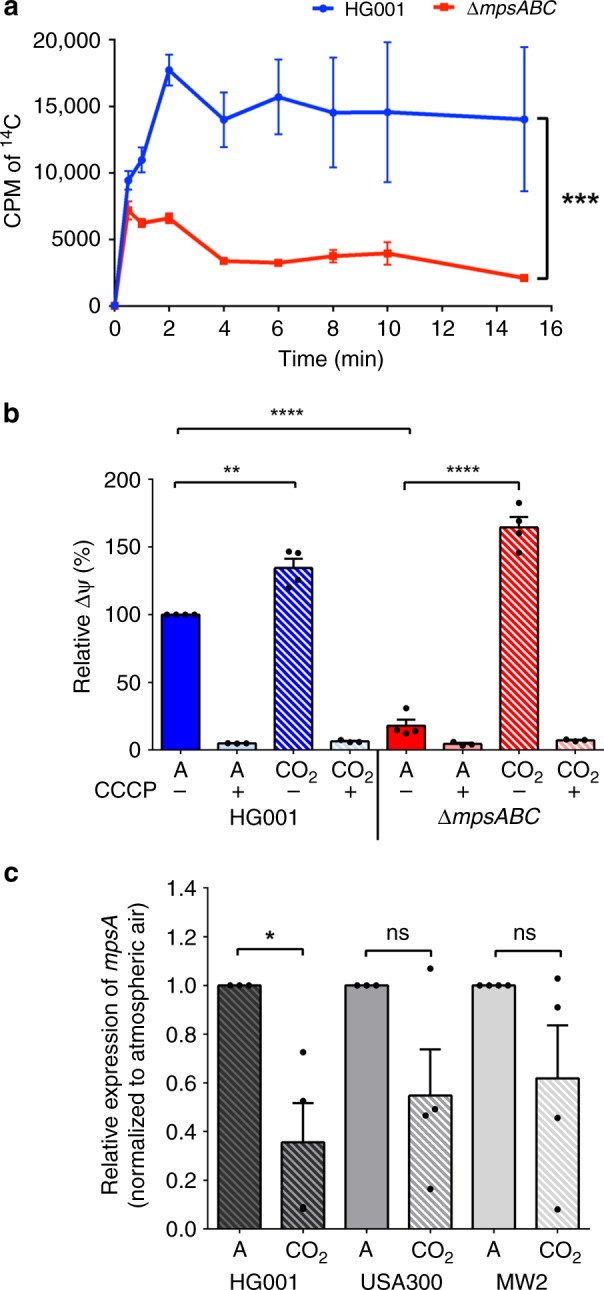
Uptake of NaH^14^CO_3_ by HG001 and Δ*mpsABC*, membrane potential determination and *mpsA* expression. **a** Cells were preincubated with fluorocitrate for 30 mins prior to the addition of NaH^14^CO_3_ (50 μCi). NaH^14^CO_3_ was added at time 0 and 1 ml of cell samples collected at times indicated. The H^14^CO_3_ uptake was determined by ^14^C accumulation in cells measured by liquid scintillation counting. The NaH^14^CO_3_ uptake in Δ*mpsABC* was significantly lower than WT HG001; ***p < 0.001 by paired two-sided *t-*test. Each point shown in the graph represents the mean value ± standard error mean (SEM) from three independent experiments. (CPM = counts per minute). **b** ∆*mpsABC* grown in 5% CO_2_ showed increased membrane potential compared with those grown in atmospheric conditions. Membrane potential was determined by fluorescence intensity measurements using BacLight bacterial membrane potential kit. The values for WT HG001 in atmospheric were set equal to 100%. Cells were grown to exponential growth phase in atmospheric (A) and 5% CO_2_ (CO_2_). As a negative control, the H^+^ ionophore CCCP caused a collapse of the membrane potential in all strains tested both in atmospheric CO_2_ and in the presence of 5% CO_2_. When grown under 5% CO_2_ conditions, HG001 and ∆*mpsABC* showed significantly higher relative membrane potential (**p < 0.01 and ****p < 0.0001, respectively, as determined by unpaired two-sided *t*-test) compared with their corresponding strains in atmospheric conditions. Each bar shown in the graph represents the mean value ± standard error mean (SEM) from four independent experiments (three independent experiments for CCCP), each with three technical replicates. **c** *mpsA* expression in three wild-type *Staphylococcus aureus* strains HG001, USA300, and MW2 under atmospheric and 5% CO_2_ conditions. The relative expression of *mpsA* under 5% CO_2_ conditions, normalized to its corresponding strains grown in atmospheric (A) conditions. Only *mpsA* expression in HG001 is significantly lower in 5% CO_2_ compared with atmospheric conditions; *p < 0.05 by unpaired two-sided *t*-test. (ns = not significant). Each bar shown in the graph represents the mean value ± standard error mean (SEM) from four independent experiments, each with three technical replicates. Source data are provided as a Source Data file

The diminished ability of the Δ*mpsABC* mutant to grow under atmospheric conditions as well as the rescue of this defect by CO_2_ and bicarbonate led us to the suggestion that MpsAB may play a role in CO_2_ and bicarbonate transport. As the determination of the CO_2_ transport is very difficult, we compared the bicarbonate uptake activity in the WT strain HG001 and the *∆mpsABC* mutant with radiolabeled NaH^14^CO_3_. Both strains were grown aerobically in TSB until reaching its respective exponential growth phase before being washed to remove the growth media and resuspended to the same OD_578_ in Tris buffer. Prior to the addition of NaH^14^CO_3_, the cell suspensions were incubated with fluorocitrate and glucose. Fluorocitrate, an aconitase inhibitor, was added to block the TCA cycle so that NaH^14^CO_3_ accumulated intracellularly and is not immediately expired by the decarboxylation reactions of the TCA cycle. Glucose was added as energy source because the cells were resuspended in only Tris buffer. After addition of NaH^14^CO_3_, samples were rapidly harvested at several time points and the ^14^C uptake was measured by liquid scintillation counting. The immediate uptake occurred within 120 s until a steady state level was reached (Fig. 3a). Compared with the WT strain HG001, *ΔmpsABC* showed significantly decreased ^14^C uptake in the first 120 s. After 15 min, Δ*mpsABC* showed a 6.6-fold lower radioactivity than the WT strain indicating that the bicarbonate uptake was impaired in the mutant.

### *mpsAB* complements an *E*. *coli* CA mutant and vice versa

**Fig. 4:**
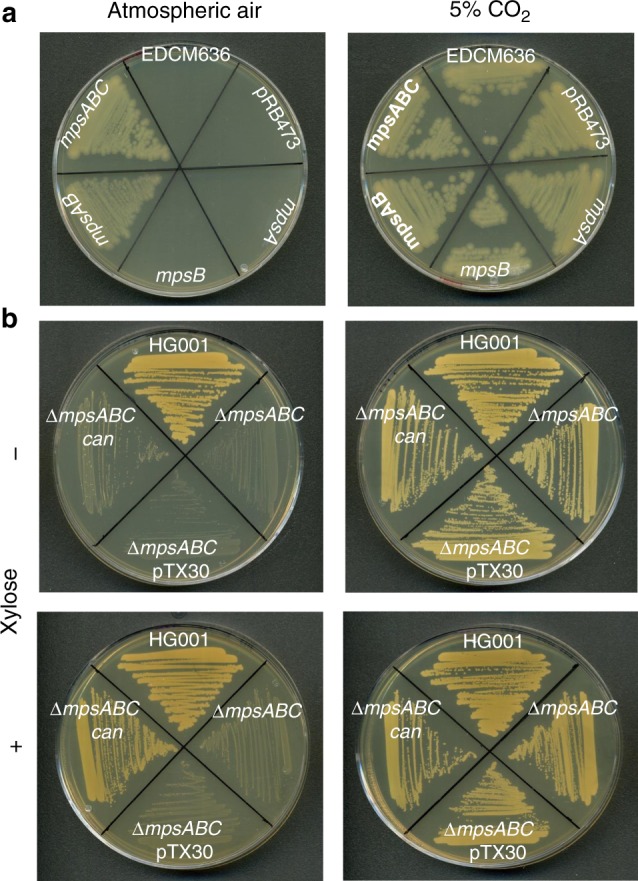
*mpsAB*(*C*) complements an *E. coli* carbonic anhydrase mutant and vice versa. **a** *E. coli* carbonic anhydrase mutant (EDCM636) can be complemented with *mpsAB* and *mpsABC* using plasmid pRB473 under the control of its native promoter in atmospheric (left) and 5% CO_2_ (right). Clockwise from the top; EDCM636, EDCM636 pRB473 (empty plasmid), EDCM636 pRB473 *mpsA*, EDCM636 pRB473 *mpsB*, EDCM636 pRB473 *mpsAB*, and EDCM636 pRB473 *mpsABC*. **b** ∆*mpsABC* can be complemented with carbonic anhydrase, *can* from *E. coli* MG1655 using pTX30, a xylose-inducible promoter plasmid. Clockwise from the top; wild-type HG001, ∆*mpsABC*, ∆*mpsABC* pTX30 (empty plasmid), and ∆*mpsABC* pTX30 *can*. Plates were supplemented with xylose (+) or without xylose (−)

Previously we described that MpsB contains a metal binding motif (His607, Cys622, and Cys625), which is reminiscent of a carbonic anhydrase (CA) motif^12^. Therefore, we tried to detect CA activity with purified MpsB alone and cell extracts expressing MpsABC. However, we were unable to detect any CA activity with various methods. In the absence of any detectable enzymatic data, we asked if *mpsAB* could complement a CO_2_-dependent *E. coli* mutant, EDCM636^18^. EDCM636 is an *E. coli* K-12 mutant in which the CA gene, *can*, was deleted. As a result, this strain is unable to grow under atmospheric air, but can grow in the presence of high CO_2_. This phenotype of EDCM636 is similar to that of HG001Δ*mpsABC*; hence, we investigated whether *mpsAB* can restore growth of EDCM636 under atmospheric conditions. To do this, we inserted the *S. aureus mpsA, mpsB, mpsAB*, and *mpsABC* genes into *E. coli* strain EDCM636. The vector alone, pRB473, served as control. Interestingly, neither *mpsA* nor *mpsB* alone could restore growth of EDCM636. Plasmids containing *mpsAB* and *mpsABC* were able to restore the growth equally well (Fig. 4a), indicating that *mpsC* was dispensable. As expected, all six strains were able to grow under 5% CO_2_ conditions (Fig. 4a).

Since *mpsAB* restored the growth of EDCM636, we asked if the CA gene (*can*) from *E. coli* could complement the *S. aureus* Δ*mpsABC*. To address this question, *can* from *E.coli* K-12 was expressed in Δ*mpsABC*(pTX30-*can*) under a xylose inducible promoter. As shown in Fig. 4b, without xylose Δ*mpsABC*(pTX30-*can*) could not grow under atmospheric air. However, when *can* expression was induced by xylose, growth was restored. All clones grew in the presence of 5% CO_2_ (Fig. 4b), suggesting that both the bicarbonate transporter and CA enable growth under atmospheric CO_2_ level and both are interchangeable. Although the activities of bicarbonate transporters and CA are different, they represent a CO_2_/bicarbonate concentrating system that can functionally replace each other.

### Impaired membrane potential in *ΔmpsABC* is rescued by 5% CO_2_

Previously, it was reported that MpsABC represents a membrane potential-generating and cation-translocating system, and that membrane potential was severely affected in the *∆mpsABC* mutant^12^. Because elevated CO_2_ restores the growth of *∆mpsABC*, we wondered whether 5% CO_2_ also restores the membrane potential in this mutant. The membrane potential of the WT strain, HG001, grown under atmospheric air was set equal to 100% and was used as a comparator for other strains (Fig. 3b). In the *ΔmpsABC* mutant, the membrane potential was decreased to only 18% of HG001. In contrast, the presence of 5% CO_2_ increased the membrane potential of the *ΔmpsABC* mutant and the WT strain up to 164% and 134%, respectively. As a negative control, the H^+^ ionophore CCCP caused a collapse of the membrane potential in all strains tested both in atmospheric CO_2_ and in the presence of 5% CO_2_. Based on these findings, elevated CO_2_ not only restores growth but also the membrane potential generation in the *ΔmpsABC*.

### *mpsA* expression was downregulated under 5% CO_2_ conditions

Since the *mps* deletion mutants, except *∆mpsC*, are responsive to 5% CO_2_, we postulated that the expression of *mpsAB* could be affected by CO_2_. To investigate whether high CO_2_ levels influence *mps* transcription, Quantitative Reverse Transcriptase-PCR (qRT-PCR) was performed to determine the transcription of *mpsA* in atmospheric and 5% CO_2_ conditions. To exclude strain specific effects, three different WT *S. aureus* strains were used, namely HG001, USA300 LAC, and MW2 (USA400). RNA was isolated during the exponential growth phase and the mRNA of *mpsA* from the three strains grown in atmospheric air and in 5% CO_2_ were compared. Transcript abundance for each strain cultivated in 5% CO_2_ was normalized to its corresponding strain grown in atmospheric conditions. Relative to the growth in atmospheric air, *mpsA* in HG001 grown in CO_2_ was downregulated the most, namely 2.5-fold, in USA300 2-fold, and in MW2 1.7-fold (Fig. 3c). In short, *mpsA* expression was lower in 5% CO_2_ compared with atmospheric conditions in all three strains tested; however, only *mpsA* downregulation in HG001 reached statistical significance (*p* < 0.05). A downregulation of *mpsA* (SAOUHSC_00412) by CO_2_ was also observed under cell culture conditions, as reported in Aureowiki^19^.

### *ΔmpsA* produces fewer hemolytic toxins and is less pathogenic

**Fig. 5:**
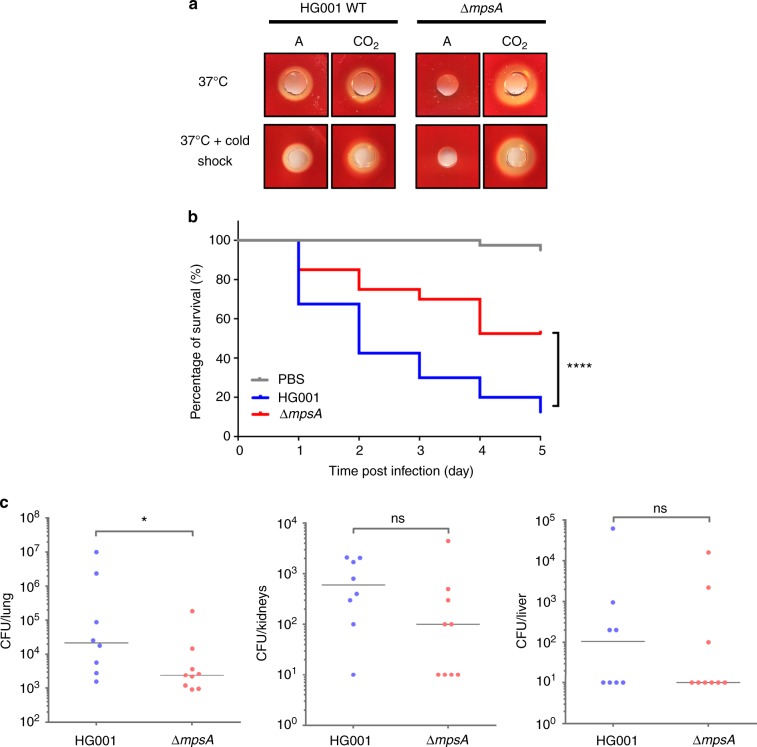
∆*mpsA* produces less hemolytic toxin in atmospheric conditions and is less virulent in animal infection models. **a** Hemolytic toxin production in HG001 and *ΔmpsA* grown in atmospheric (left) and 5% CO_2_ (right) at 37 °C after overnight incubation (above) and after cold shock treatment (below). **b** Kaplan–Meier plot of the survival of *Galleria mellonella* larvae infected with HG001 and *ΔmpsA*. Larvae injected with PBS were used as control. Larvae infected with *ΔmpsA* showed significantly better survival rate compared with the larvae infected with wild-type HG001; ****p < 0.0001 as determined by Log-rank (Mantel–Cox) test. Results are representative of four independent experiments. **c** Comparison of wild-type HG001 and *ΔmpsA* in a 48 h intranasal mouse infection model (pneumonia model) in the lung, kidneys, and liver. The bacterial burden of *ΔmpsA* in the lung was significantly lower (*p = 0.036) compared with HG001. There was no significant difference (ns) in bacterial burden in the kidney and liver between the two strains. The number of bacteria per organ based on determination of CFU in *n* = 8 (WT HG001) and *n* = 9 mice (*ΔmpsA*) is shown. The vertical line indicates the median. The detection limit was 10 CFU per organ. Data were analyzed using two-tailed Mann–Whitney test. Source data are provided as a Source Data file

Because the CO_2_/bicarbonate transporter MpsAB is important for growth and membrane potential generation, we predicted that the mutant would have less toxin production and be less virulent. To examine this question, the hemolytic activity of *ΔmpsA* and HG001 grown in atmospheric and 5% CO_2_ was tested on sheep blood agar followed by cold shock treatment. Consistent with our hypothesis, no visible hemolytic zone was seen in *ΔmpsA* when cultivated in atmospheric air, while the presence of 5% CO_2_ restored toxin production as indicated by a clear halo on blood agar (Fig. 5a). The same result was also observed for the *ΔmpsB* and *ΔmpsABC* mutants (Supplementary Fig. 4). The WT strain HG001 formed a hemolysis zone independent of the presence of 5% CO_2_.

Following the finding of decreased toxin production from *ΔmpsA*, we were interested to find out if this effect is relevant in vivo. To characterize the role of *mps* on virulence, *Galleria mellonella* larvae were used as an invertebrate infection model. The survival rate of larvae injected with WT HG001, *ΔmpsA*, and PBS as control were observed over the course of 5-day period (Fig. 5b). In the PBS control group, 95% of the larvae were alive at the end of the 5-day period. In contrast, larvae infected with HG001 showed a survival rate of only 13% after the 5-day period, while 53% of the larvae survived when infected with *ΔmpsA*. These results indicate that deletion of *mpsA* rendered *S. aureus* less virulent in an invertebrate infection model.

To assess the contributions of *mpsAB* to *S. aureus* pathogenesis using an intranasal mouse infection model (pneumonia model), female Balb/c mice were intranasally infected with HG001 and *ΔmpsA*. After 48 h, mice were sacrificed and the lungs, kidneys, and livers were recovered to determine the bacterial burden. Although the *ΔmpsA* generally exhibited a lower bacterial burden in all three organs, only the bacterial load in the lung was significantly lower compared with its WT strain HG001 (Fig. 5c). These results indicate that the *ΔmpsA* is attenuated in pathogenicity compared with its WT strain in this mouse infection model. Taken together, these findings suggest that a functional bicarbonate transport system (mps) is required for fitness and pathogenicity in vivo.

### Phylogenetic distribution of *mpsAB* homologs

**Fig. 6:**
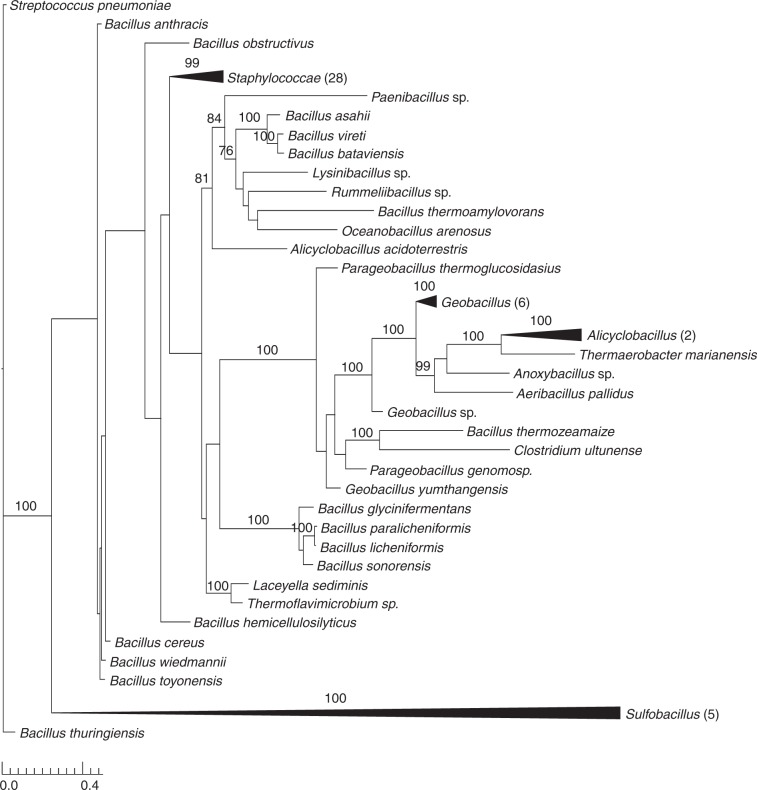
*mpsAB* homologs are widespread in the *Firmicutes* phylum. Maximum likelihood phylogenetic analysis of concatenated alignments of *mpsA* and *mpsB* homologs amongst the phylum *Firmicutes.* Node support is indicated by bootstrap values from 100 resamplings of the alignment when they exceeded 70%. Collapsed clades are labeled by the genus of associated taxa. The number of taxa belonging to the respective clade is indicated in brackets. Source data are provided as a Source Data file

Considering the significant effect of *mpsAB* in *S. aureus*, we examined the phylogenetic distribution of both genes. Searching the RefSeq database revealed genes homologous to *mpsA* and *mpsB* were found in a number of species that span the *Firmicutes* phylum (Fig. 6). These included bacteria from the genus *Staphylococcus, Bacillus* and a few from *Alicyclobacillus, Geobacillus, Parageobacillus, Sulfobacillus*, and *Thermaerobacter* (Fig. 6 and Supplementary Fig. 6). Furthermore, homologs of *mpsAB* were also widespread in genomes from multiple phyla of prokaryotes (Supplementary Fig. 7). Among those which were well depicted in the phylogenetic tree are from the phyla *Proteobacteria, Actinobacteria*, and *Bacteroidetes*. MpsAB are highly conserved in the genus *Staphylococcus* and to a lesser extent in *Bacillus* and other *Firmicutes* (Supplementary Fig. 5). Apart from few exceptions in *Actinobacteria* and *Proteobacteria*, in most genomes *mpsA* and *mpsB* homologs are always adjacent. In *Acidimicrobium ferrooxidans*, a ferrous-iron-oxidizing bacterium, mpsA and mpsB homologs exist as a single fused gene (Supplementary Fig. 5).

Apart from *S. aureus*, *mpsAB* homologs were found in a few human pathogens such as *Bacillus anthracis*, *Legionella pneumophila*, and *Vibrio cholerae* (Table 1). Interestingly, all these three pathogens also carry genes encoding protein with CA domain in addition to *mpsAB* homologs. In the family of *Staphylococcaceae*, only *Macrococcus caseolyticus* JCSC5402, *Staphylococcus carnosus* TM300, and *Staphylococcus pseudintermedius* ED99 do not have *mpsAB* homologs. Instead, they carry genes with CA homologs. This surprising observation also extend to a number of human pathogens; *Listeria monocytogenes, Streptococcus pyogenes, Enterococcus faecalis, Enterococcus faecium, Mycobacterium tuberculosis, Helicobacter pylori, Escherichia coli, and Pseudomonas aeruginosa* (Table 1). All these important pathogens have CA homologs instead of *mpsAB* homologs, emphasizing the importance of bicarbonate/CO_2_ supply to the microorganisms.

**Table 1:** Distribution of MpsAB and carbonic anhydrase (CA) homologs in various species

Species	MpsAB homolog	CA homolog
*Staphylococcus aureus aureus* NCTC 8325	+	−
*Staphylococcus carnosus carnosus* TM300	−	+^(a)^
*Staphylococcus pseudintermedius* ED99	−	+^(a)^
*Macrococcus caseolyticus* JCSC5402	−	+^(a)^
*Bacillus anthracis* delta Sterne	+	+^(a)^
*Bacillus subtilis subtilis* 168	+	+ +^(a)^
*Listeria monocytogenes R479a*	−	+^(b)^
*Streptococcus pneumoniae* R6	−	+^(a)^
*Streptococcus pyogenes* A20	−	+^(a)^
*Enterococcus faecalis* V583	−	+^(b)^
*Enterococcus faecium* DO	−	+^(b)^
*Mycobacterium tuberculosis* H37Rv	−	+ + +^(a)^*
*Helicobacter pylori* J99	−	+^(a)^ +^(b)^
*Escherichia coli* O157:H7 Sakai (EHEC)	−	+ +^(a)^
*Legionella pneumophila pneumophila* Philadelphia-1	+	+ + +^(a)^*
*Pseudomonas aeruginosa PAO1*	−	+ + +^(a)^
*Vibrio cholerae* sv. O1 bv. El Tor N16961	+	+^(a)^ +^(b)^

Homologies were inferred based on PFam domains search from finished bacterial genomes in Integrated Microbial Genomes & Microbiomes (IGM/G) database. MpsA, MpsB, prokaryotic type-CA, and eukaryotic-type CA belongs to PFam00361, PFam10070, PFam00484, and PFam00194, respectively. The symbol +/− indicates the presence or absence of homolog, while (a) and (b) indicates prokaryotic and eukaryotic-CA, respectively. Asterisk indicates that one of the three CAs is a gene fusion and has a probable transmembrane domain in which the N-terminal part has PFam00916 (sulfate transporter family) and PFam00484 in the C-terminal part

## Discussion

Despite an almost 50-year-old observation of CO_2_-dependent *S. aureus* growth, little is known about the genetic basis of *S. aureus* regarding its response to changes in CO_2_ availability. In the present work, we demonstrate that the growth defect of the *ΔmpsABC* mutant under atmospheric CO_2_ levels could be largely rescued by 5% CO_2_ (Fig. 2a) or by ≥ 50 mM bicarbonate (Fig. 2b). Uptake studies with radiolabeled NaH^14^CO_3_ indicates that MpsABC contributed to a significant accumulation of H^14^CO_3_^−^ in the cytoplasm of the WT strain (Fig. 3a). The bicarbonate uptake experiment is tricky because we had to prevent CO_2_ evaporation as much as possible. This is most likely the reason why we observed increased variations after 5 min. However, one should consider that for substrate uptake, the first 2 min are crucial. During this time range, the standard error mean (SEM) values were really small. We assume that the addition of fluorocitrate was probably not inhibiting the TCA cycle completely; we do not dare to increase the concentration any higher because of the side effects it may cause (we used 10 mM fluorocitrate and the normal concentration used for aconitase enzyme assay is 100 μM).

Deletion of the *mpsC* gene showed no phenotypic alterations or growth defect, suggesting it is not a functional part of the *mpsAB* operon. Furthermore, the *E. coli* CA mutant could be complemented with only *mpsAB*, and the gene synteny revealed that *mpsC* is not collocated with *mpsAB* homologs in other bacteria than staphylococci. Taken together, these observations speak in favor of MpsAB representing a bicarbonate or DIC transporter with the ability to accumulate intracellular DIC.

CAs are Zn^2+^ metalloenzymes, which catalyze the reversible hydration of CO_2_ (CO_2_ + H_2_O ↔ HCO_3_^−^ + H^+^). For this reason, we hypothesized that a CA mutant in *E. coli* (EDCM636) could be complemented by *mpsAB*. The *E. coli can* gene encodes a β-class CA, which is essential under atmospheric but not at 5% CO_2_^18^. As expected, the *E. coli can* mutant could be complemented by *mpsAB* and vice versa; the *S. aureus mpsABC* mutant could be complemented by *E. coli* specific *can* (Fig. 4). Due to the mutual complementation, we thought that MpsB, MpsAB, or MpsABC might have CA activity. Unfortunately, we were unable to detect CA activity using purified proteins or with whole cell extracts. The absence of CA activity in MpsAB suggests that MpsAB and CA have different enzymatic mechanisms, yet they serve the same function; namely, preventing evaporation of CO_2_ by trapping it as bicarbonate for anaplerotic metabolism. Although a considerable amount of CO_2_ is produced by a functional TCA cycle, this CO_2_ will be lost in the cytoplasm due to an insufficient concentration for anaplerotic reactions. To prevent this from happening, CA catalyzed the formation of bicarbonate from CO_2_, which significantly delays the release of intracellular CO_2_ and providing bicarbonate required for carboxylation reactions^20^.

**Fig. 7:**
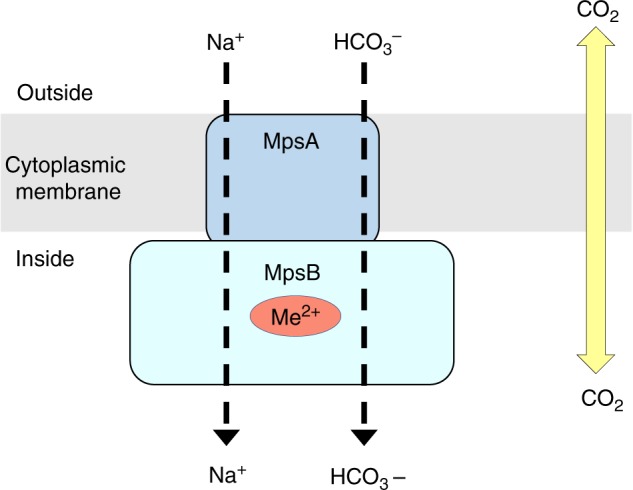
Proposed function for MpsAB. We proposed that MpsAB represents a sodium bicarbonate cotransporter (NBC). These transporters are prevalent in mammalians and are expressed throughout the body. Unlike CO_2_ which can diffuse passively in and out of the cells, bicarbonate (HCO_3_^−^) needs to be transferred into the cells via a transporter. In *S. aureus*, MpsAB most likely function together as cotransporter to transport Na^+^ and HCO_3_^−^. Me^2+^ indicates a metal binding site in MpsB

The observation that EDCM636 could only be complemented by both MpsAB suggests they function together as a transporter. This is also supported by the collocation of mpsAB homologs in many microorganisms across multiple phyla (Fig. 6, Supplementary Figs. 5 and 7). Furthermore, MpsA belongs to PFam00361^21^, which consists of a diverse family of proton-conducting membrane transporters, including NADH:quinone oxidoreductase subunits (complex I)^22^. In this regard, we demonstrated that MpsABC constitutes a cation-translocating system, capable of Na^+^ transport similar to the observation regarding NuoL^12^. MpsB on the other hand belongs to PFam10070, a family of conserved proteins in bacteria of unknown function. Based on these observations, we propose a model that MpsAB is a sodium bicarbonate cotransporter (NBC) as illustrated in Fig. 7. Such NBC transporters are prevalent in mammals^23^, having important functions in maintaining intracellular and whole-body pH, as well as contributing to the transepithelial transport processes. The bicarbonate cotransporters are composed of 13 transmembrane domains^24^, similar to MpsA which has 14. It is unknown whether mammals also require an MpsB homolog; although the NBC stoichiometry in *Xenopus* oocytes is 1 Na^+^: 2 HCO_3_^−^, indicating a possible accessory protein interaction^25^. As CO_2_ is volatile, transporting bicarbonate or DIC helps to “trap” it in the cytoplasm for the carboxylation reactions of anaplerotic metabolism. In *Firmicutes*, these include pyruvate carboxylase fuelling the TCA cycle, acetyl-CoA-carboxylase as the first enzyme of fatty acid synthesis, or phosphoribosylaminoimidazole carboxylase involved in purine biosynthesis.

Phylogenetic analysis revealed that *mpsAB* are widespread, not only among *Firmicutes*, but also across multiple phyla (Fig. 6 and Supplementary Fig. 7). Bacteria that possess homologs of MpsAB have diverse lifestyles; for example hyperthermophilic (e.g., *Aquifex aeolicus*), thermophilic and acidophilic (e.g., *Acidimicrobium ferrooxidans* and *Acidithiobacillus caldus*), CO_2_ fixing bacteria (e.g., *Chloroflexus aurantiacus)*, and nitrogen-fixing bacteria (e.g., *Bradyrhizobium oligotrophicum* and *Frankia sp*). In addition, MpsAB homologs were also described in the gammaproteobacterial chemolithoautotroph *Thiomicrospira crunogena*^15^. Similar to *S. aureus*, the *T. crunogena* genes *Tcr_0853* (*mpsA* homolog) and *Tcr_0854* (*mpsB* homolog) were cotranscribed, constitute a probable DIC transporter, and its mutants required elevated DIC for growth^15^. Although *T. crunogena* has CO_2_ concentrating mechanisms (CCMs), its DIC uptake process is unclear due to the lack of cyanobacterial transporter homologs. That being said, it is assumed that in autotrophic bacteria, a DIC concentrating system is necessary to ensure that Rubisco is saturated with CO_2_, so that carboxylation proceeds at its maximum rate.

Another problem with the “ancient” Rubisco is that it also reacts with oxygen^26^. In this respect, the atmospheric CO_2_ content in ancient times was high, while that of O_2_ was low. In a CO_2_-rich environment, the CO_2_-fixing enzymes in autotrophic bacteria, such as Rubisco could work properly despite its low affinity and limited discrimination between CO_2_ and O_2_. However, due to oxygenic photosynthesis in the subsequent 2.5 billion years, the atmosphere became O_2_ rich and CO_2_ poor^27^. As a consequence, the cytoplasmic CO_2_ levels became too low for proper CO_2_ fixation and assimilation^28,29^. Therefore, it is assumed that autotrophic bacteria that grow in atmosphere appear to uniformly have CCMs^27^. Bacterial CCM involves two main functions: (a) active transport of inorganic carbon species (HCO_3_^−^ and CO_2_) to concentrate HCO_3_^−^ within the cell, and (b) the pool of HCO_3_^−^ is then utilized to provide elevated CO_2_ concentrations around primary CO_2_ fixing enzyme, Rubisco, encapsulated in carboxysomes which also contain CA^14^. Recently, DIC transporters from four evolutionary distinct families were described in several genera of sulfur-oxidizing chemolithoautotrophs^16^. Two of these DIC transporters demonstrate DIC uptake even though they share low protein identity with the well-characterized cyanobacterial transporters, SbtA- and SuIP-family bicarbonate transporters. SbtA is an inducible, high-affinity Na^+^-dependent HCO_3_^−^ transporter^30^, while BicA, a member of the SuIP family, is a low-affinity, high-flux Na^+^-dependent HCO_3_^−^ transporter^31^. Heterologously expressed chromate ion transporter from *Hydrogenovibrio thermophilus* JR2 is also capable of DIC uptake^16^. The fourth type of transporter is a two component DIC transporter encoded by the genes *Tcr_0853* and *Tcr_0854* in *Hydrogenovibrio crunogenus* (previously known as *T. crunogena)* as discussed previously^15,16^. Another well-characterized DIC transporter expressed in cyanobacteria is BCTI, encoded by the *cmpABCD* operon is an inducible, high-affinity HCO_3_^−^ transporter belonging to the ATP binding cassette transporter family^32^. It is thought that these transporters differ in their affinities and mechanisms for transport (as cation symporter or anion antiporter), which provide advantages under specific growth conditions^16^. Out of all these distinct transporters families, MpsAB belongs to the same type as encoded by *Tcr_0853* and *Tcr_0854* from *H. crunogenus* in terms of function and homology; MpsAB is 29–30% identical to *Tcr_0853* and *Tcr_0854*. Such transporters and their function were so far not described in *Firmicutes*.

Staphylococci, like most other *Firmicutes*, are neither autotrophic nor do they possess typical carboxysome structures or Rubisco. The question is, therefore, why do they need MpsAB as a DIC concentrating system. The most rational answer would be that enzymes involved in substrate carboxylation are not sufficiently supplied with HCO_3_^−^ under atmospheric CO_2_, thus require a higher HCO_3_^−^ concentration for proper activity. For example, the biotin-dependent pyruvate carboxylase has a central function in fueling the TCA cycle with oxaloacetate. If this reaction is impaired, not only the TCA cycle but also respiration and the membrane potential would be impaired. This is exactly the observed phenotype of the *mpsABC* mutant, which exhibited the typical features of SCV in atmospheric CO_2_ and similar to the *S. aureus hemB* mutant^10,12^. It is also tempting to speculate that MpsAB serve to prevent the depletion of cellular DIC, a role which is conventionally played by CA or other known cyanobacterial bicarbonate transporter. *S. aureus* has neither of them, however since CO_2_ or HCO3^−^ is crucial for many cellular processes, a DIC concentrating mechanism is likely to be important for its growth and survival. As a major pathogen *S. aureus* needs to adapt to different niches; as commensal inhabitant on environmentally exposed skin and mucosal surfaces to colonization and infection inside the mammalian host. Variations of CO_2_ levels are one of the evident conditions it would encounter in this process, and having a DIC concentrating mechanism would ensure its sufficient supply intracellularly. Collectively, these hypotheses could explain the role of MpsAB beyond the known carbon-fixing bacteria.

Due to the interchangeable role of MpsAB and CA, we sought to compare the occurrence of both the genes in *Firmicutes*. In this phyla, there is tendency that either one of them is present; in some cases both genes are present in one strain/species, for example in *B. anthracis* and *B. subtilis* (Table 1). This same observation appears to hold true for selected species of Gram-positive and Gram-negative pathogens (Table 1). Interestingly, highly pathogenic species for example *Legionella pneumophila* and *Vibrio cholera* have MpsAB homologs as well as at least two CA homologs, while *Mycobacterium tuberculosis, Helicobacter pylori*, *E. coli* O157:H7, and *Pseudomonas aeruginosa* have two or even three CAs. The importance of the distribution of MpsAB and CA in the respective strains or species is not yet clear. Nevertheless, the occurrence of two and even three CA genes, and/or MpsAB suggests that DIC concentrating mechanism play an important role in fitness and pathogenicity. We assume that the decreased virulence of the *mpsA* mutant is due to the very low intracellular bicarbonate levels. Bicarbonate is the substrate for biotin carboxylase that produces carboxybiotin, the substrate for other carboxylases, such as PEP−, or pyruvate carboxylase that feed the TCA cycle. For example, the *K*_m_ value for bicarbonate of the *E. coli* biotin carboxylase is comparatively high at 16 mM^33^, indicating that the enzyme has a very low affinity for bicarbonate. This suggests that a high level of intracellular bicarbonate is needed to fuel the TCA cycle, respiration, fatty acid biosynthesis, and membrane potential. ATP and membrane potential are important for toxin secretion.

The virulence attenuation of the *ΔmpsA* was also seen in vivo. In the *Galleria mellonella* larvae survival model, the killing rate by the mutant was significantly decreased (Fig. 5b). In the intranasal *S. aureus* mouse infection model (pneumonia model), the CFU/lung was also significantly decreased with *ΔmpsA* (Fig. 5c). We have chosen a lung infection model because the CO_2_ content in the lung is higher and we expect that the *mpsA* mutant might have a better chance to grow. CO_2_ is produced during respiration and is carried by the blood through the venous system to the lungs, where it is exhaled with a concentration of ~3.8% in the tidal air or 450 l of CO_2_ per day^34^. There was no statistically significant difference in bacterial burden in the kidney and liver between HG001 and the *mpsA* mutant. This can be explained that the lung was the primary organ that was infected with *S. aureus*. From there, *S. aureus* spreads to other organs during the course of infection. Spreading is a complex process as the bacteria have several barriers to pass: first the lung epithelium and endothelium of blood vessels, and then again the endothelium of blood vessels to establish infections in other organs. Thus, infection burden in kidneys and liver was lower than in the lung, as it has also been observed by Lee et al.^35^.

In conclusion, we propose that MpsAB constitutes a DIC transporter that is important for growth of *S. aureus* under low, atmospheric CO_2_ conditions. This transporter system underlies the importance of CO_2_/bicarbonate in the fitness and pathogenicity of *S. aureus* and may help shed some light on earlier observations about CO_2_-dependent variants. Furthermore, *mpsAB* homologs are widespread in diverse groups of microorganisms that adopt different lifestyles and occupy a wide range of niches. In contrast to the dogma of DIC transporters, MpsAB plays an important function even in non-autotrophic bacteria by concentrating bicarbonate for anaplerotic pathways that are relevant across most microorganisms. An interesting finding in our study was that MpsAB and CA could functionally replace one another in our mutual complementation experiments, further supporting the essentiality of bicarbonate. Within this context, a number of the pathogenic species have either MpsAB or CA, whereas both are present in some of these species, suggesting their roles in growth and virulence.

## Methods

### Bacterial strains and growth conditions

The strains used in this study are listed in Supplementary Table 1. For cloning procedures, all staphylococcal and *E. coli* strains were cultivated at 37 °C with shaking at 150 rpm in basic medium (BM) (1% soy peptone, 0.5% yeast extract, 0.5% NaCl, 0.1% glucose, and 0.1% K_2_HPO_4_, pH 7.2), unless specified otherwise. All cultures were grown in 10 ml medium using baffled 100 ml shake flasks (flask-to-medium ratio 10:1) as recommended^36^, with the exception of growth studies, which were grown in 15 ml medium (flask-to-medium ratio 7:1). The medium was supplemented with the following antibiotics, where applicable at the indicated final concentrations: chloramphenicol at 10 μg ml^−1^, tetracycline at 25 μg ml^−1^, for staphylococcal strains and 100 μg ml^−1^ ampicillin and 30 μg ml^−1^ kanamycin for *E. coli* strains.

### Growth studies of staphylococcal strains

TSB (Sigma-Aldrich) and TSA plates (Sigma-Aldrich) were used for growth studies involving *S. aureus*. For growth characterization on solid medium, *S. aureus* strains were precultured for 24 h at 37 °C under continuous shaking starting from a single colony. Cultures were then diluted to 10^−5^–10^−7^ and an aliquot of 100 μl was plated on TSA plates. The plates were incubated for 5 days at 37 °C under atmospheric and 5% CO_2_ conditions. The 5% CO_2_ level was achieved in a CO_2_ incubator (Heraeus Instruments). The colony size and appearance on TSA was observed every 24 h for 5 days and documented with a Leica M125 stereomicroscope. For growth characterization in liquid medium, *S. aureus* cells were precultured in TSB as described above. *S. aureus* strains involved were WT HG001, Δ*mpsA*, Δ*mpsB*, Δ*mpsC*, and Δ*mpsABC* that were transformed with pRB473-*mpsA*, pRB473-*mpsB*, pRB473-*mpsC*, and pRB473-*mpsABC*, respectively. Main cultures were inoculated to OD_578_ = 0.05 and grown under atmospheric and 5% CO_2_ conditions at 37 °C under continuous shaking. Aliquots were taken at 2, 4, 6, 8, 10, 12, 24, 48, and 72 h for OD_578_ measurements.

### Growth studies of Δ*mpsABC* with bicarbonate supplemented media

Media were supplemented with NaHCO_3_. Precultures were grown as described above. Main cultures were cultivated as described above as well, but with the addition of the supplements at final concentrations of 1, 5, 10, and 50 mM and grown under atmospheric conditions. Δ*mpsABC* grown under atmospheric and 5% CO_2_ conditions were used as controls.

### Complementation experiments of Δ*mpsABC* and EDCM636

With regards to *S. aureus* Δ*mpsABC* complemented with *can* from *E. coli*, the cultures were inoculated and were adjusted to OD_578_ = 5, then the strains were streaked on agar plates containing no xylose or 0.5% xylose (filter sterilized). Complementation of EDCM636 with pRB473-*mpsA*, pRB473-*mpsB*, pRB473-*mpsAB*, and pRB473-*mpsABC*, were also performed as described above, with the exception that BM and BMA were used instead of TSB and TSA.

### Construction of the *S*. *aureus* deletion mutants

All oligonucleotides used in this study are listed in Supplementary Table 2. Nucleotide and amino acid sequences were obtained from the Kyoto Encyclopedia of Genes and Genomes (KEGG). The deletion mutants *S. aureus* HG001 Δ*mpsB* (KEGG accession no. SAOUHSC_00413) and Δ*mpsC* (SAOUHSC_00414) were constructed as markerless deletions using allelic replacements as described by Bae and Schneewind^37^. Briefly, 1 kb upstream and 1 kb downstream of *mpsB* and *mpsC*, respectively were amplified from the chromosomal DNA of *S. aureus* HG001. The fragments were assembled with linearized (Bg1II restriction enzyme) plasmid pBASE6^38^ by Gibson assembly^39^ using Hi-Fi DNA Assembly Master Mix (New England Biolabs). The resulting plasmids were first introduced into *E. coli* DC10B^40^ and then into *S. aureus* RN4220 via electroporation before transformation of *S. aureus* HG001. Deletion of the genes were confirmed by PCR and sequence analysis. For *S. aureus* HG001 Δ*mpsA* and Δ*mpsABC*, pKOR1^37^ carrying up- and downstream flanking regions of ∼2 kb each for both deletions^12^ were used.

### Construction of complementation vectors

Complementation of Δ*mpsB* was carried out with plasmid pCtufamp^41^ and Δ*mpsC* with plasmid pRB473^42^. For construction of complementation vector for *mpsB*, *mpsB* was amplified and then assembled into linearized pCtufamp plasmid (HindIII and PacI restriction enzyme) by Gibson assembly. As for construction of complementation vector for *mpsC*, the putative native promoter sequence for *mpsABC* as well as *mpsC* were amplified and then inserted into linearized pRB473 (EcoRI restriction enzyme). The putative promoter regions were determined by DNA sequence analysis. The constructed plasmids were first introduced into *E. coli* DC10B then into *S. aureus* RN4220 before being transformed into *S. aureus* HG001 Δ*mpsB* and Δ*mpsC*, respectively. For complementation of Δ*mpsA* and Δ*mpsABC*, recombinant vector plasmids pRB473-*mpsA* and pRB473-*mpsABC* from previous study^12^ were isolated and the corresponding deletion mutants were transformed as described above. In addition, all constructed pRB473 derivatives were transformed into EDCM636, an *E. coli* MG1655 derivative harboring a kanamycin resistance marker replacing a deletion of the CA encoding gene *can*. It was purchased from *E. coli* Genetic Stock Center, Yale University. Additional complementation vectors pRB473-*mpsB* and pRB473-*mpsAB* were constructed and transformed into EDCM636, including the empty vector.

### Construction of pTX30-*can*

The xylose inducible plasmid pTX30^43^ was used for expression of *can* (KEGG accession no. b0126). Therefore, *can* was amplified from the chromosomal DNA of *E. coli* MG1655. The insert was inserted into the linearized pTX30 (BamH1 and EcoR1 restriction enzymes) using T4 DNA ligase (Thermo Scientific). The resulting plasmids was transformed into *S. aureus* RN4220 via electroporation before being transformed into Δ*mpsABC*.

### Quantitative RT-PCR

qRT-PCR was carried out to determine the gene expression of *mpsA* in three WT *S. aureus* strains, namely HG001, USA300^44^, and MW2^45^ under atmospheric and 5% CO_2_ conditions. All bacteria precultures were precultivated in atmospheric and 5% CO_2_ conditions, respectively, for 24 h. Main cultures were inoculated at an OD_578_ = 0.1 for each strain and grown in its respective growth conditions for 3 hrs. Subsequently an aliquots of OD_578_ = 2.0 were harvested. RNA isolation was carried out using a RNeasy Mini kit (Qiagen) according to manufacturer’s instructions. The resulting RNA was treated with RQ1 RNase-Free DNase (Promega). Subsequently, the RNA was analyzed for DNA contamination using ReproFast DNA Polymerase (Genaxxon Bioscience) with no-RT control PCR. Power SYBR^®^ Green RNA-to-CT™ 1-Step Kit (Applied Biosystems) was used in AviaMx Real-Time PCR System (Agilent Technologies) with primers directed to *mpsA* using 50 ng of RNA as template in each reaction. DNA gyrase subunit B (*gyrB*) was used as housekeeping gene control. The RNA used in qRT-PCR was isolated from four independent experiments. The relative expression of *mpsA* under 5% CO_2_ was normalized to its corresponding strains grown in atmospheric conditions.

### Bicarbonate uptake analysis

Main cultures were inoculated to an OD_578_ = 0.1 and grown until exponential growth phase for 3 h (*S. aureus* HG001) and 18 h (Δ*mpsABC*). Cells were then centrifuged and washed with 10 mM Tris buffer at pH 7. Cells were resuspended in Tris buffer and adjusted to OD_578_ of 1 in a final volume of 10 ml. Fluorocitrate and glucose at a final concentration of 10 mM and 5 mM, respectively, were added to both cell suspensions before incubated at room temperature with magnetic stirring for 30 min. After that, 50 μCi of NaH^14^CO_3_ (specific activity 58 mCi mmol^−1^, Hartmann Analytic) was added. One milliliter of samples was collected at time 0 (before the addition of NaH^14^CO_3_), 0.5, 1, 2, 4, 6, 8, 10, and 15 min. After each sample was collected, cells were immediately filtered by vacuum filtration onto membrane filters (Whatman^TM^ ME 25 mixed cellulose ester membrane filters 0.45 μm) and washed with 10 ml of Tris NaCl buffer (10 mM Tris with 100 mM NaCl). The membrane filters were then placed in a vial containing 10 ml of liquid scintillation cocktail (Ultima Gold, PerkinElmer). Radioactivity retained on the membrane filters was taken as H^14^CO_3_ uptake which was determined by ^14^C accumulation in cells, measured by liquid scintillation counting (LKB Wallac 1209 RackBeta liquid scintillation counter). Results were recorded as counts per minute (CPM).

### Hemolysis assay

To analyze hemolytic toxin production, overnight cultures of HG001 Δ*mpsA*, Δ*mpsB*, and Δ*mpsABC* grown in 5% CO_2_ were used. Each bacterial strain was adjusted to an OD_578_ of 2 and streaked onto blood agar (Oxoid). In addition, to better visualize the hemolysin halo zones, cultures were grown in atmospheric (16 h for HG001 and 48 h for Δ*mpsA*) and in 5% CO_2_ conditions (16 h for both HG001 and Δ*mpsA)*. An aliquot of same OD_578_ was collected for each strain before being centrifuged to remove the cell pellets. The remaining supernatant was filtered and concentrated with SpeedVac. The concentrated supernatant was then dropped into blood agar. All plates were incubated at 37 °C under atmospheric and 5% CO_2_ conditions respectively for 24 h before subjected to cold shock treatment for 72 h.

### *Galleria mellonella* larvae infection model

Final-stage instar larvae of *G. mellonella* were purchased from Reptilienkosmos.de, Germany. Ten larvae weighing between 300 and 600 mg were infected with *S. aureus* HG001 and Δ*mpsA*, respectively. Bacteria were grown in TSB at 37 °C with shaking for 24 h (HG001) and 48 h (Δ*mpsA*). Cells were washed twice and resuspended in Dulbecco’s Phosphate-Buffered Saline (DPBS) (Gibco^TM^) before being adjusted to an OD_578_, which corresponds to 5 × 10^8^ colony-forming unit (CFU) for each strain. Each larva was injected with 5 × 10^6^ cells. Larvae injected with DPBS served as control group. The larvae were incubated at 37 °C for 5 days after infection and surviving larvae were counted every day starting from 24 h after infection. The experiments were replicated four times. Data collected from each independent experiment were pooled for statistical analysis, resulting in *n* *=* 40 for every strain.

### Membrane potential measurement

Main cultures were grown to exponential growth phase for HG001 (3 h in atmospheric and 5% CO_2_, respectively) and also for Δ*mpsABC* (18 h in atmospheric and 3 h in 5% CO_2_ conditions). Cells were washed once in phosphate-buffered saline (PBS) before adjusted to an OD_578_ of 0.4. Membrane potential was determined using BacLight bacterial membrane potential kit (Invitrogen) according to the manufacturer’s protocol. Briefly, 10 μl of the dye 3,3′-diethyloxacarbocyanine iodide (DiOC2[3]) was added to the bacterial suspension samples and incubated for 30 min. Then, 200 μl of the stained or unstained sample was applied to a black, flat-bottomed 96-well plate to determine the red and green fluorescence intensity using a microplate reader (Tecan Infinite M200). Excitation and emission wavelengths were chosen as recommended by the kit’s protocol. The fluorescence intensities of wild-type HG001 grown in atmospheric was set equal to 100%.

### Mouse infection experiments

Overnight cultures of HG001 and *ΔmpsA* in Brain Heart Infusion Broth (BHI) medium were diluted to a final OD_600_ of 0.05 in 50 ml fresh BHI medium and grown until exponential growth phase (3.5 h for HG001 and 6 h for *ΔmpsA)* at 37 °C with 5% CO_2_. After centrifugation, the cell pellet was resuspended in BHI with 20% glycerol, aliquoted and stored at −80 °C. For the generation of in vivo infection, aliquots were thawed and washed twice with PBS. The infectious dose used for infection was very similar for both strains (2.1 × 10^8^ CFU/HG001 and 1.7 × 10 CFU/*ΔmpsA* in 20 μl). A sample of the infection inoculum was plated on TSB agar plates in order to control the infection dose. For the intranasal *S. aureus* infection model (pneumonia model) we used female Balb/c mice (8 or 9 per group, 6 weeks, Janvier Labs, Le Genest-Saint-Isle, France). They were intranasally infected with 2.1 × 10^8^ CFU of HG001 and with 1.7 × 10^8^ CFU of *ΔmpsA*. During infection, mice were scored twice a day and the severity of infection was determined accordingly to recognize if humane endpoint was reached. After 48 h of infection, mice were sacrificed, the lungs, kidneys, and livers recovered, homogenized and plated in serial dilutions on TSB agar plates incubated overnight at 37 °C with 5% CO_2_ in order to determine the bacterial burden. Significant difference in the CFU counts in the organs between the two groups was determined with Mann–Whitney test.

### Ethics statement

All of the animal studies were approved by the local government of Lower Franconia, Germany (approval number 55.2-2532-2-155) and performed in strict accordance with the guidelines for animal care and experimentation of German Animal Protection Law and the DIRECTIVE 2010/63/EU of the EU. The mice were housed in individually ventilated cages under normal diet in groups of four to five throughout the experiment with ad libitum access to food and water.

### Phylogenetic analysis of *mpsA* and *mpsB* homologs

Homologs of *mpsA* (YP_498998.1) and *mpsB* (YP_498999.1) from *S. aureus* were identified from the RefSeq database^46^ using Microbial Protein BLAST. All searches were either conducted within the whole Bacteria domain, or restricted to the bacterial phylum Firmicutes or the bacterial genus Staphylococcus. Since several species have more than one putative homologs of *mpsA* and *mpsB*, we restricted the hits only to those protein pairs of *mpsA* and *mpsB*, whose coding sequences, extracted from the respective GenBank files, are in immediate proximity in the genome. The protein sequences of *mpsA* and *mpsB* homologs were aligned separately using Clustal Omega (version 1.2.1)^47^. The multiple sequence alignments (MSAs) were subsequently concatenated into one single MSA comprising 350 taxa (Bacteria), 83 taxa (*Firmicutes*), and 29 (*Staphylococcus*), respectively. Phylogenetic trees were constructed using maximum likelihood (ML) analysis provided by the program RAxML (version 8.2.9)^48^. The GAMMA Model of rate heterogeneity was used and all model parameters were estimated by RAxML. LG (Bacteria, *Staphylococcus*) and JTT (*Firmicutes)* with empirical base frequencies were determined to be the best-scoring (likelihood score) protein substitution model. Results were assessed using 100 bootstrap replicates. The best tree was visualized using TreeGraph2 (version 2.14.0–771 beta)^49^.

### Occurrence of MpsAB and CA homologs based on PFam domains

The occurrence of MpsAB and CA homologs were inferred based on PFam domains^21^ search from finished bacterial genomes in Integrated Microbial Genomes & Microbiomes (IGM/G) database^50^. MpsA, MpsB, prokaryotic type-CA, and eukaryotic-type CA belongs to PFam00361, PFam10070, PFam00484, and PFam00194, respectively. Results were presented as the presence (if yes, the frequency it was found in a particular species) or absence of the respective PFam domains.

### CA assays

CA assay was performed using purified N-terminal strep-tagged MpsB protein. Two methods were used to detect CA activity, electrometric and colorimetric methods. The electrometric method refers to enzymatic assay of CA for Wilbur-Anderson Units (EC 4.2.1.1), as listed in the website of Sigma-Aldrich (document CR BIOT-MAJ-1013), based on cited references^51–53^. One Wilbur-Anderson unit will cause the pH of a 20 mM Trizma buffer to drop from 8.3 to 6.3 per minute at 0 °C. The assay is briefly described here and the full protocol can be found on Sigma-Aldrich’s website^54^. Assay buffer was prepared at 20 mM using Trizma Base and purified water (Milli-Q) and the pH was adjusted to 8.3 with 2 N Sulfuric Acid. CO_2_ saturated solution was used as substrate and was prepared by placing a small piece of dry ice into cold Milli-Q water for at least 30 min prior to the experiment. CA enzyme standard solution was used as control. It was prepared fresh prior to the experiment by dissolving 2 mg of CA from bovine erythrocytes lyophilized powder, ≥2000 W-A units mg^−1^ protein (Sigma-Aldrich, Germany). Then the concentrated standard solution was diluted to 60–90 U ml^−1^. This concentration corresponds to a reaction time between 10 and 20 s. The buffer reference standards (pH 4,7, and 10) and pH electrode to equilibrate to <3 °C before calibrating the pH meter with each of the reference buffer. All solutions and dram vials were kept cold on ice prior to use. To perform the blank reaction, 3 ml of ice cold assay buffer and 0.05 ml of ice cold purified water were pipetted into a dram vial with a micro stir bar. The temperature of the reaction mixture was checked with thermometer and proceed to the next step if the temperature was <3 °C, by placing the pH electrode in the solution, with stirring. After the pH had reached >8.5, 2 ml of ice cold CO_2_ saturated solution was added. The time required for the pH to change from 8.3 to 6.3 was recorded. This step was repeated a few times and once the blank time of 65 s was reached, the experiment proceeded immediately with CA control. For control, 3 ml of ice cold assay buffer was added to the dram vial and if the temperature was <3 °C and subsequently when the pH > 8.5, 2 ml of ice cold CO_2_ saturated solution was added to the reaction mixture. When the pH reached 8.4–8.5, 0.05 ml of CA standard solution was added. The time required for the pH to change from 8.3 to 6.3 was recorded. If the time is not in the 10–20 s range, a more concentrated enzyme standard must be prepared. If the blank average time (from a minimum of five blank values) was in the range of 70–100 s, the experiment could proceed with purified MpsB protein. The steps for MpsB protein were the same as control, in which 0.05 ml of MpsB was used instead of CA standard solution. The CA U ml^−1^ is determined as:

$${\mathrm{Units}}\;{\mathrm{ml}}^{ - 1}\;{\mathrm{enzyme}}:\frac{{\left( {{{T}}_{{\mathrm{blank}}\,{\mathrm{average}}}-{{T}}_{{\mathrm{sample}}\,{\mathrm{average}}}} \right){\mathrm{/df}}}}{{\left( {{{T}}_{{\mathrm{sample}}\;{\mathrm{average}}}} \right)\left( {0.05} \right)}}$$

where df = Dilution factor

*T *= Time (in seconds) required for the pH to change from 8.3 to 6.3 as per the Unit Definition

0.05 = Volume (in milliliter) of enzyme used

We found that this method is unreliable because of the difficulty in maintaining the temperature at <3 °C. Therefore, inconsistencies in the pH readings and subsequently the deviations in the time for the pH change from 8.3 to 6.3 resulted in inaccurate and non-reproducible readings. Therefore, we attempted another assay using colorimetric method. The colorimetric method to detect CA activity was performed according to previous studies^20,55^ with slight modifications. The assay was based on changing-pH/dye indicator method using a Tecan Infinite M200 injector system. Briefly, two reaction buffers were prepared: (a) a buffer using phenol red as indicator (200 mM), 50 mM HEPES, and 200 mM Na_2_SO_4_ at pH 6.5, 7, and 7.5, (b) a buffer using m-cresol purple as indicator (200 mM), 50 mM TAPS, and 200 mM Na_2_SO_4_ at pH 7.5 and 8.4. CO_2_ saturated water was prepared by placing a small piece of dry ice in Milli-Q water for at least 30 mins prior to the experiment. Purified MpsB protein was diluted in buffers and the reaction was initiated by adding an equivalent amount of CO_2_ saturated water with the Tecan injector system. The subsequent color changes of the pH-sensitive dye indicators were monitored by Tecan microplate reader every 0.2 s for 1 min at 558 nm (pH 6.5–7.5) or 578 nm (pH 7.5–8.4). All reactions were performed at room temperature (~25 °C) and in a final volume of 1 ml. The CA activities (if any) of MpsB protein and positive control CA from bovine erythrocytes lyophilized powder (Sigma-Aldrich, Germany) were measured at final concentrations of 100 and 0.1 μg ml^−1^, respectively. A total of 50 mM Tris-HCl at corresponding pH of the buffers was used as nonenzymatic controls. Due to the nondetectable CA activity, we repeated the assay with whole cell lysate of wild-type HG001, *ΔmpsB*, and *ΔmpsABC* with and without addition of NADH and finally with resting cell suspensions of the three strains. However, no detectable CA activity was observed.

### Statistical analyses

Data are presented as mean values and SEM from at least three independent biological replicates unless specified otherwise. Data from the mouse experiment are presented as median values. Normal distributions were analyzed by Student's *t*-test. The larvae infection model was analyzed using Log-rank (Mantel–Cox) test. For the mouse infection experiments, two-tailed Mann–Whitney-test was employed to compare the difference of CFU counts in the organs between the mutant clones with the WT HG001. All statistical analyses were performed using GraphPad Prism 6.0 software. A *p* value of <0.05 was considered to be statistically significant, with *n* represents independent biological replicates.

### Reporting summary

Further information on research design is available in the Nature Research Reporting Summary linked to this article.

## Supplementary information

Supplementary Information

Reporting Summary

Source Data

## Data Availability

The source data underlying Figs. 1a, 2, 3, 5b & c, 6 and Supplementary Figs. 2, 3, 6, and 7 are provided as a Source Data file. Other data supporting the findings of this work are available within the paper and its Supplementary Information files, or from the corresponding author upon request.
