# Supplementary material for: MpsAB is important for Staphylococcus aureus virulence and growth at atmospheric CO2 levels
Source: Nat Commun. 2019 Aug 9;10:3627. doi: 10.1038/s41467-019-11547-5 (PMC6689103; doi:10.1038/s41467-019-11547-5)
Supplement: Supplementary file 1 — Supplementary Information [file 41467_2019_11547_MOESM1_ESM.pdf]

Supplementary Information

MpsAB is important for *Staphylococcus aureus* virulence and growth under atmospheric CO_2 levels

Fan et al.

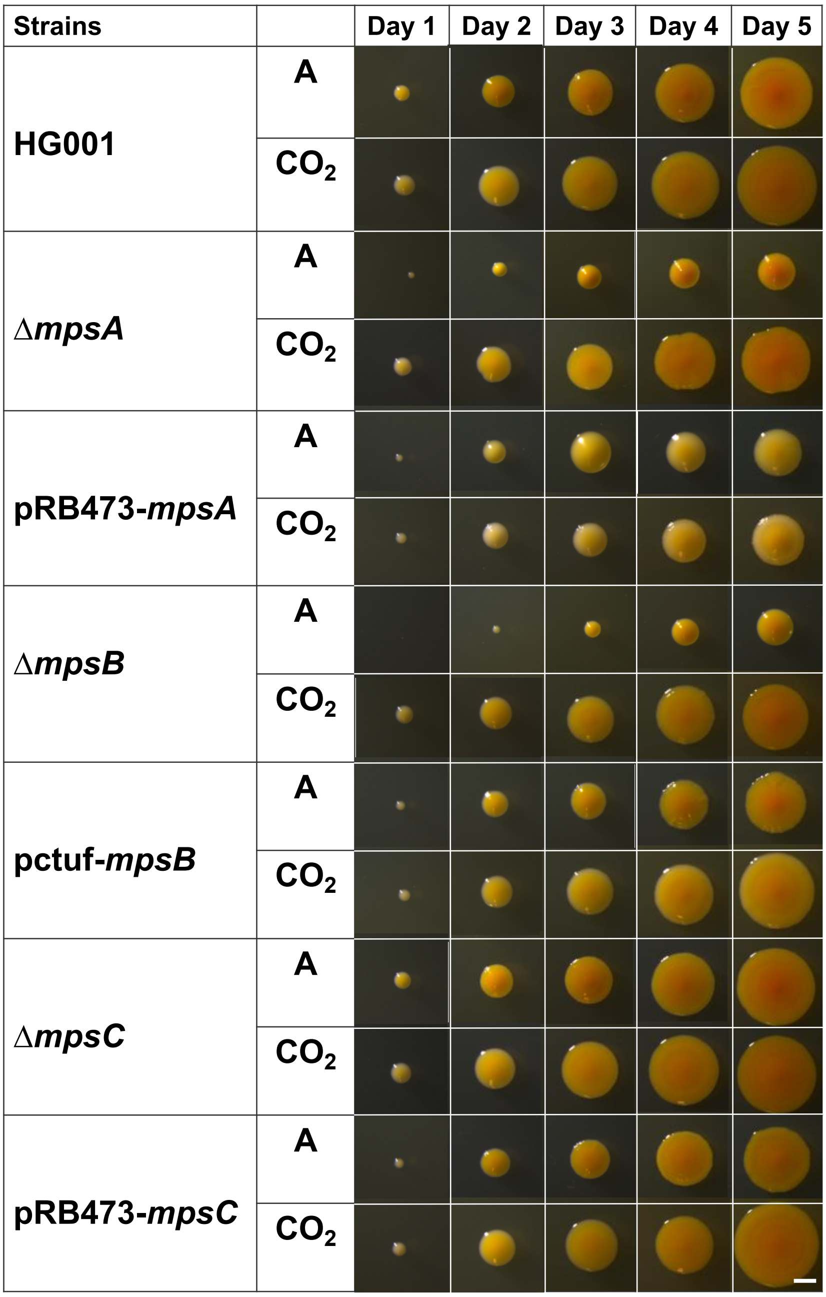

Supplementary Figure 1: Growth of HG001 *ΔmpsA, ΔmpsB* and *ΔmpsC*
Colony appearance of wild type HG001, *ΔmpsA, ΔmpsB* and *ΔmpsC* and its corresponding complemented mutant during five days of incubation in atmospheric conditions and 5% CO_2. White bar represents a scale of 1 mm.

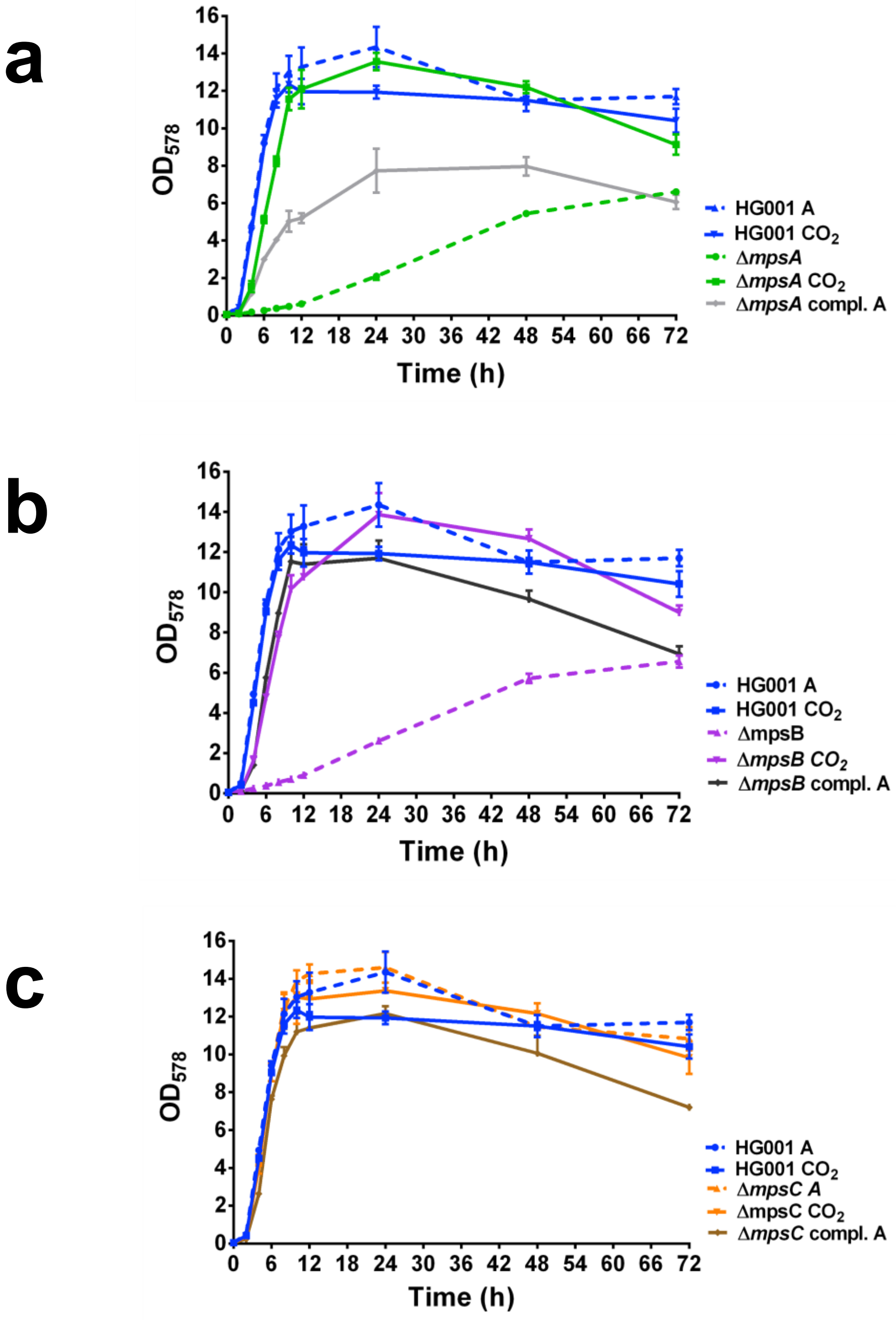

Supplementary Figure 2: Growth of HG001 *mps* deletion mutants

Growth of HG001, (a) Δ*mpsA*, (b) Δ*mpsB* and (c) Δ*mpsC* and their corresponding complemented mutant in atmospheric conditions (A) and 5% CO_2. The growth of the deletion mutant *mpsA* and *mpsC* can also be complemented by plasmid pRB473 carrying *mpsA* and *mpsC* respectively. The growth of deletion mutant *mpsB* was complemented by plasmid pctuf carrying *mpsB*. Each point shown in the graph represents the mean value ± standard error mean (SEM) from three independent experiments. Source data are provided as a Source Data file.

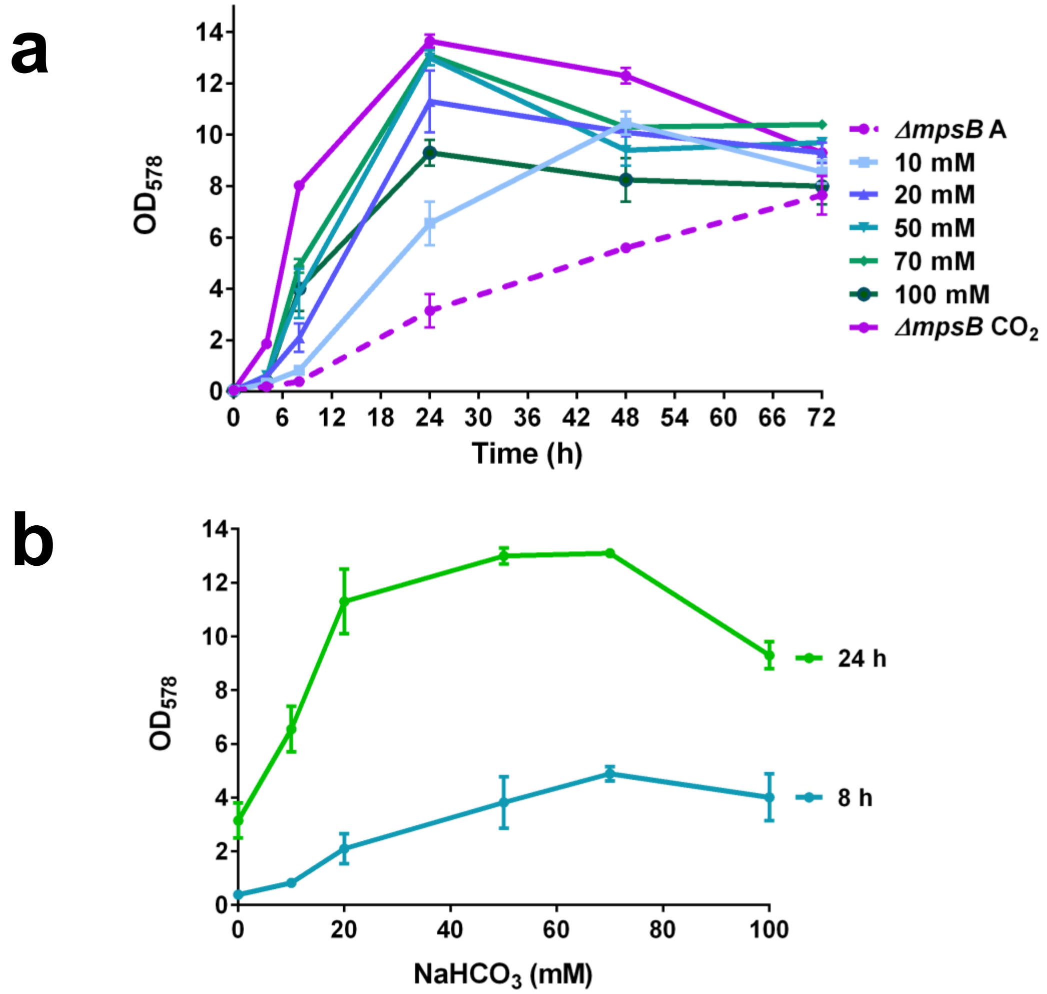

Supplementary Figure 3: Growth of HG001 Δ*mpsB* can be complemented with $NaHCO_3$

(a) Growth of Δ*mpsB* in atmospheric (A) and with additions of $NaHCO_3$ and also 5% CO_2. Each concentration of $NaHCO_3$ is indicated in the graph, ranging from 10 mM to 100 mM. Additions of $NaHCO_3$ improved the growth of Δ*mpsB* in a concentration dependent manner up to 70 mM. Each point shown in the graph represents the mean value ± standard error mean (SEM) from two independent experiments.

(b) The same graph illustrated with OD as a function of bicarbonate concentration at 8 h and 24 h. Source data are provided as a Source Data file.

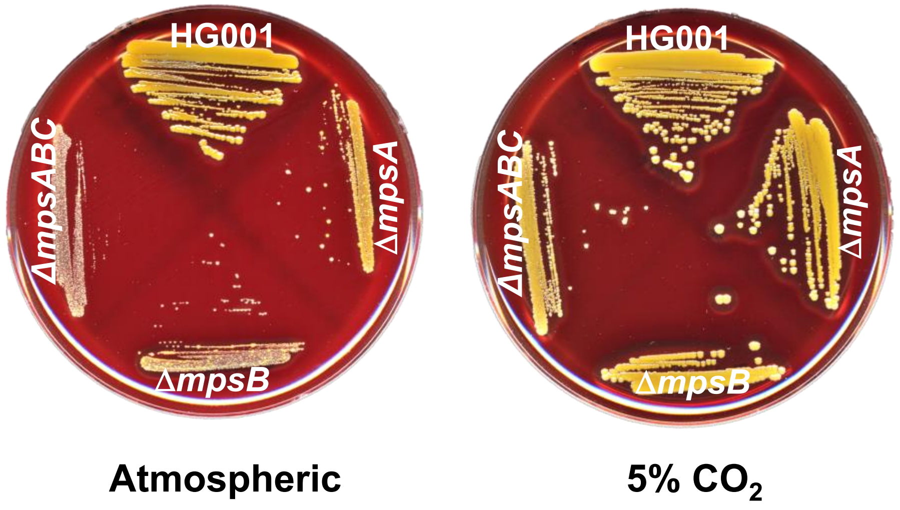

Supplementary Figure 4: The production of hemolytic toxin in HG001 and its three *mps* mutant strains (*ΔmpsA, ΔmpsB* and *ΔmpsABC)* grown under atmospheric and 5% CO_2 conditions.

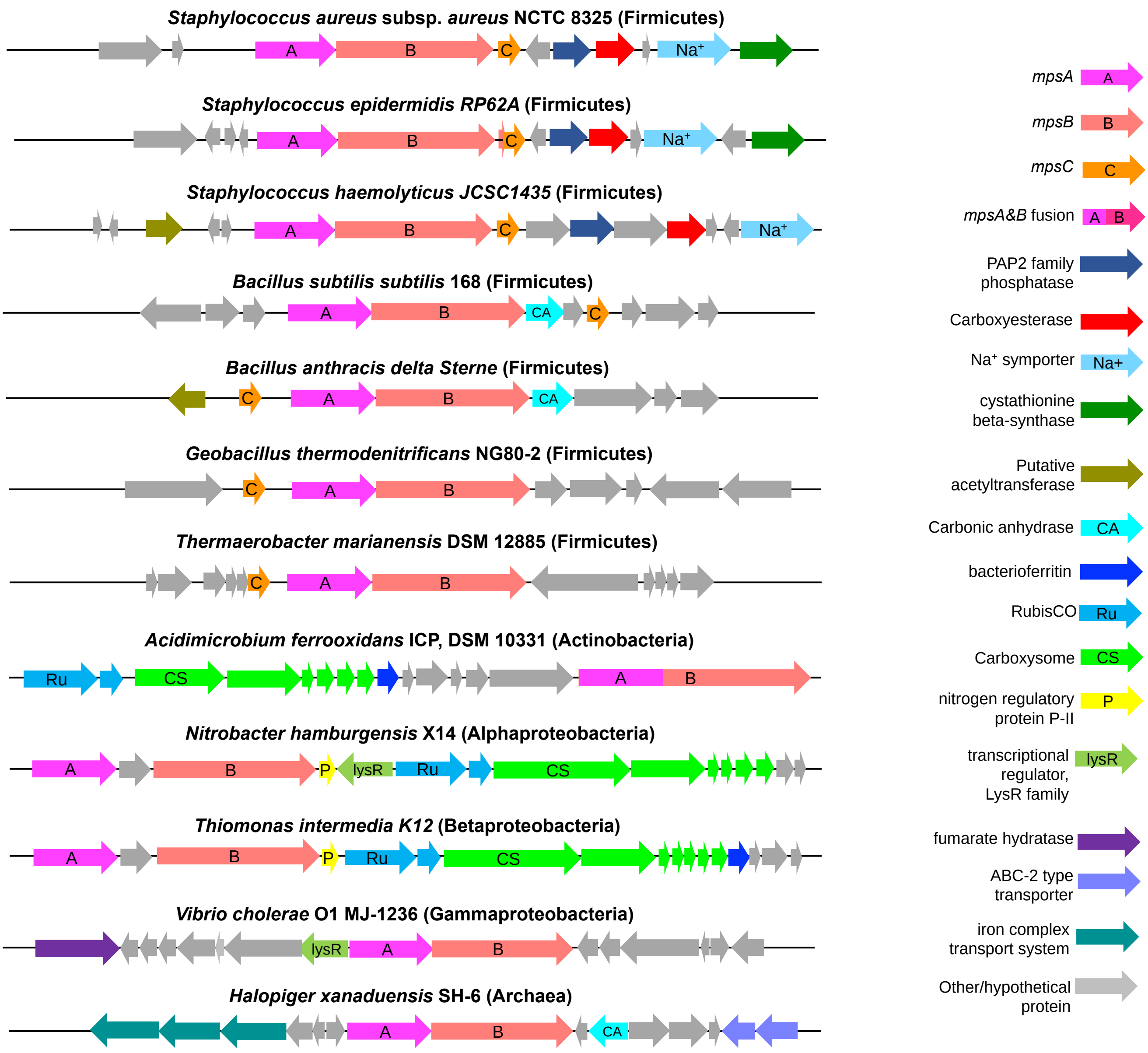

Supplementary Figure 5: Gene synteny presenting the genome organization around *mpsAB* in *S. aureus* and its homologs in selected genomes
mpsAB homologs are almost always collocated. Homologs of *mpsAB* exists as a single fused gene in *Acidimicrobium ferrooxidans*. *mpsAB* always points to the right, even if it is located on the complementary strand.

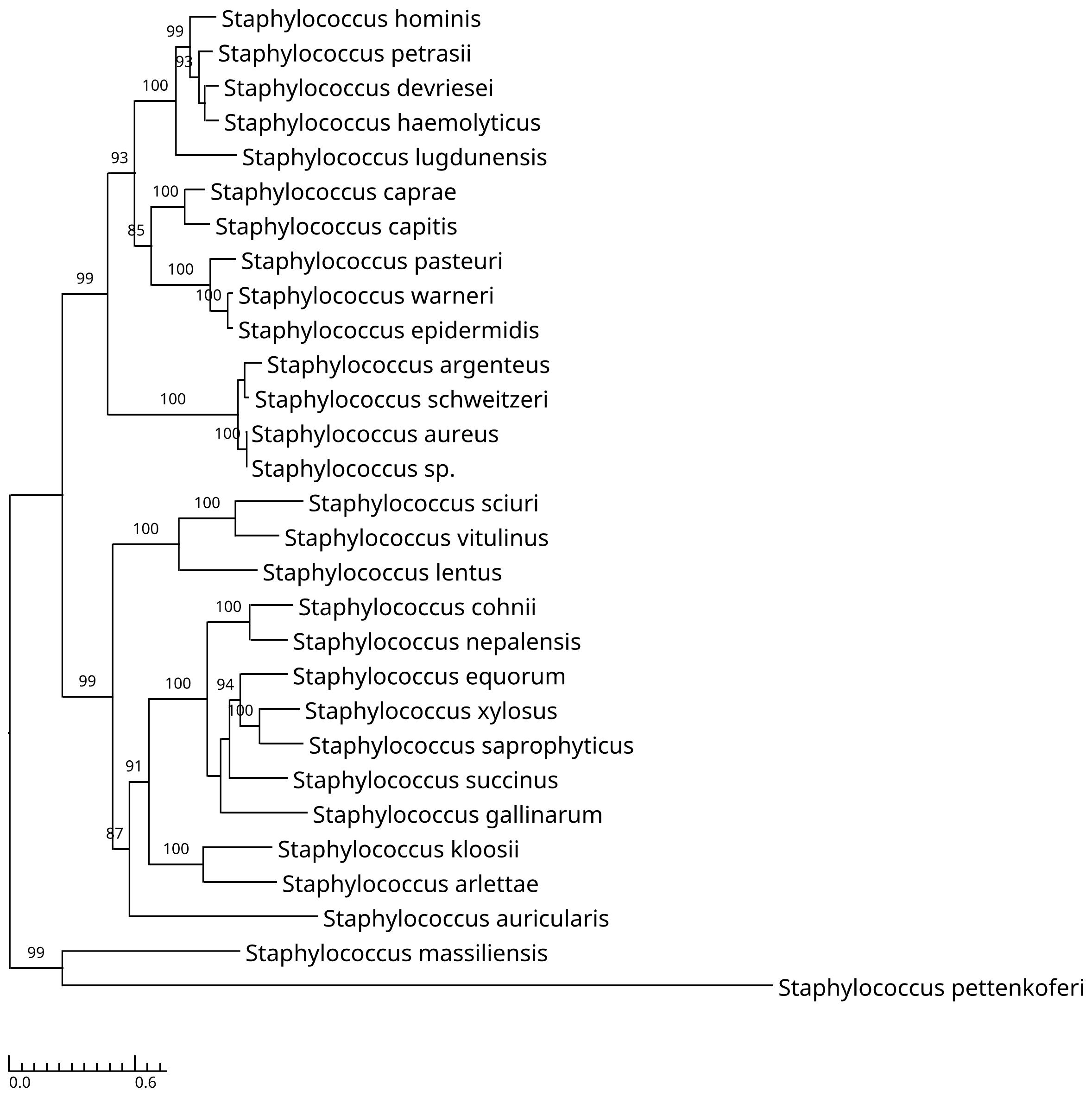

Supplementary Figure 6: Phylogenetic tree of Staphylococci
Maximum likelihood phylogenetic analysis of concatenated alignments of *mpsA* and *mpsB* homologs amongst the genus Staphylococcus. Node support is indicated by bootstrap values from 100 resamplings of the alignment when they exceeded 70%. Source data are provided as a Source Data file.

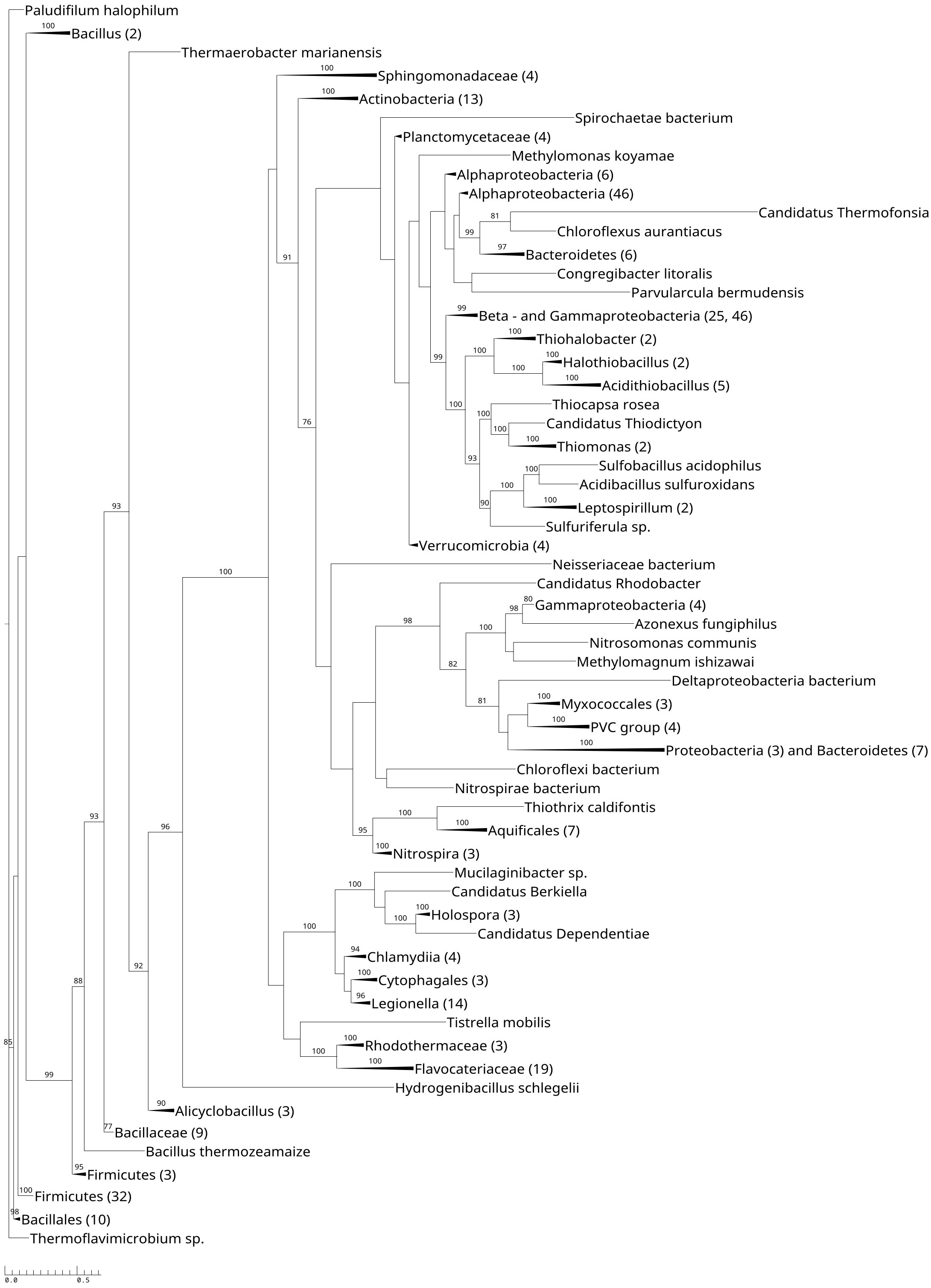

Supplementary Figure 7: Phylogenetic tree of all bacterial phyla

Maximum likelihood phylogenetic analysis of concatenated alignments of *mpsA* and *mpsB* homologs amongst the bacterial domain. Node support is indicated by bootstrap values from 100 resamplings of the alignment when they exceeded 70%. Collapsed clades are labeled by the shared taxonomic rank of associated taxa. The number of taxa belonging to the respective clade is indicated in brackets. Source data are provided as a Source Data file.

Supplementary Table 1: Bacterial strains used in this study

Strain	Description	Reference or source
E.coli EDCM636	*E. coli* MG1655 (Δ*canA1*:: *FLK2(lacZ,kan),* λ^-, *Δfnr-267,rph-1)* replacement of *can* by kanamycin resistance marker	[1]
E.coli EDCM636 *mpsA*	*E.coli* EDCM636 (pRB473-*mpsA*) carrying *mpsA*	This study
E.coli EDCM636 *mpsB*	*E.coli* EDCM636 (pRB473-*mpsB*) carrying *mpsB*	This study
E.coli EDCM636 *mpsAB*	*E.coli* EDCM636 (pRB473-*mpsAB*) carrying *mpsA* and *mpsB*	This study
E.coli EDCM636 *mpsABC*	*E.coli* EDCM636 (pRB473-mpsABC) carrying *mpsA, mpsB* and *mpsC*	This study
S. aureus HG001	NCTC8325 derivative with repaired *rsbU*	[2]
S. aureus HG001 Δ*mpsA*	Δ*mpsA* (deletion of SAOUHSC_00412)	This study
S. aureus HG001 Δ*mpsB*	Δ*mpsB* (deletion of SAOUHSC_00413)	This study
S. aureus HG001 Δ*mpsC*	Δ*mpsC* (deletion of SAOUHSC_00414)	This study
S. aureus HG001 Δ*mpsABC*	Δ*mpsABC* (deletion of SAOUHSC_00412, SAOUHSC_00413, SAOUHSC_00414)	This study
S. aureus HG001 Δ*mpsA* (pRB473-*mpsA*)	*S. aureus* HG001 Δ*mpsA* complemented with *mpsA*	This study
S. aureus HG001 Δ*mpsB* (pctuf-*mpsB*)	*S. aureus* HG001 Δ*mpsB* complemented with *mpsB*	This study
S. aureus HG001 Δ*mpsC* (pRB473-*mpsC*)	*S. aureus* HG001 Δ*mpsC* complemented with *mpsC*	This study
S. aureus HG001 Δ*mpsABC* (pRB473-*mpsABC*)	*S. aureus* HG001 Δ*mpsABC* complemented with *mpsABC*	This study
S. aureus HG001 Δ*mpsABC* (pTX30-can)	*S. aureus* HG001 Δ*mpsABC* with insertion of carbonic anhydrase, *can* from *E.coli* MG1655)	This study
S. aureus USA300	Community-associated methicillin resistant clinical isolate, pulsed- field type USA300	[3]
S. aureus USA400(MW2)	Community-associated methicillin resistant clinical isolate, pulsed- field type USA400	[4]

Supplementary Table 2: Oligonucleotides used in this study

Primer name	Sequence (5'→3')
For the construction of *S. aureus* HG001 Δ*mpsB*	
mpsB_up_fwd	CCGGGCTAGCGCGCAGATCTGTAACTATTTATATTGCGACTG
mpsB_up_rev	CCAACTTTTTAACTGTGTTGTCATACCTTTC
mpsB_down_fwd	ACAACACAGTTAAAAAGTTGGATTTAACGATGATATG
mpsB_down_rev	GCTTGATATCGTCGACAGATCTTATTTTTGTCTATATAGCGTTAATCATTAC
For the construction of *S. aureus* HG001 Δ*mpsC*	
mpsC_up_fwd	GGTACCCGGGCTAGCGCGCAGATCTCAAGCGGTTTTAATGCTAAG
mpsC_up_rev	TATGCTTTTCTTTCGTTCTTTTCATATCACTAAC
mpsC_down_fwd	AAGAACGAAAGAAAAGCATATTTAAGAAGGTTAGG
mpsC_down_rev	CAAGCTTGATATCGTCGACAGATCTCCAAACCGCTAAATGCCTC
For the construction of *S. aureus* HG001 Δ*mpsB* complementation plasmid	
pc_mpsB_fwd	GTTCGAGGAGGTTTAATTAATGACAACACAGTTAAATATCAATTC
pc_mpsB_rev	TAAGTACTTCAGCTAATTAAGCTTAAATCCAACTTTTAAAACGCC
For the construction of *S. aureus* HG001 Δ*mpsC* complementation plasmid	
pmspA_com_fwd	ATCCCCGGGTACCGAGCTCGAATTCTTATTTTAAAAGGACGGGAAATAC
pmpsA_com_rev	ACTAACTCCATATTTCTAATCTCTCGCATAATTG
mpsC_com_fwd	GATTAGAAATATGGAGTTAGTGATATGAAAAGAAC
mpsC_com_Rev	CAAGCGCTCATCGCAGTGCGAATTCATTCATCCACGTAATAAACAC
For the construction of plasmids pRB473-*mpsAB* and pRB473-*mpsB*	
PmpsABC Fwd	CCTGCAGGTCGACTCTAGAGGATCCGGACGGGAAATACTGCCTAAC
PmpsABC Rev	TTCACCTCCTTATTTCTAATCTCTCGCATAATTGCTTATG
SD+mpsB Fwd	AGAGATTAGAAATAAGGAGGTGAAAGGTATGAC
SD+mpsB Rev	TCGCAGTGCGAATTCGAGCTCTTAAATCCAACTTTTAAAACGC
For the construction of plasmid pTX30-*can*	
BglII_SD_can_fwd	AATGTAAGATCTAGGAGGTTTAATTAATGAAAGACATAGATACACTCATCAG
can_rev_EcoRI	ATTATAGAATTCATATTATTTGTGGTTGGCGTGTTTCAG
For qRT-PCR	
mpsA qpcr Fwd	TTTGCAGTCATTGGCTTTTTCGGT
mpsA qpcr Rev	AAGATGCAAACGACGTAATCGCAGT
gyrB Fwd	AAGGTATTATGGCGGCACGTG
gyrB Rev	ATCGCCTGCGTTCTAGAGTCAC

Supplementary Note 1

For Figure 2b in the manuscript, the pH levels were checked at the beginning of the experiment. The pH of the medium (TSB) increased with increased concentration of $NaHCO_3$, as shown in the Supplementary Table 3.

Supplementary Table 3: pH of medium with addition of $NaHCO_3$

Supplementation	pH of medium (TSB) at the beginning
Aerobic	7.173
1 mM $NaHCO_3$	7.199
5 mM $NaHCO_3$	7.306
10 mM $NaHCO_3$	7.380
50 mM $NaHCO_3$	7.782
CO_2	7.156

In addition, we checked the effect of pH on the growth of Δ*mpsABC* (see figures below). For this reason, we used $NaHCO_3$ at 20 mM which gave an initial pH of 7.582. As can be seen from Supplementary Figure 8, the growth of the Δ*mpsABC* in CO_2 and $NaHCO_3$ were similar (left), although the pH for the medium with $NaHCO_3$ was higher (right), indicating that pH of the medium does not play a crucial role in promoting the growth.

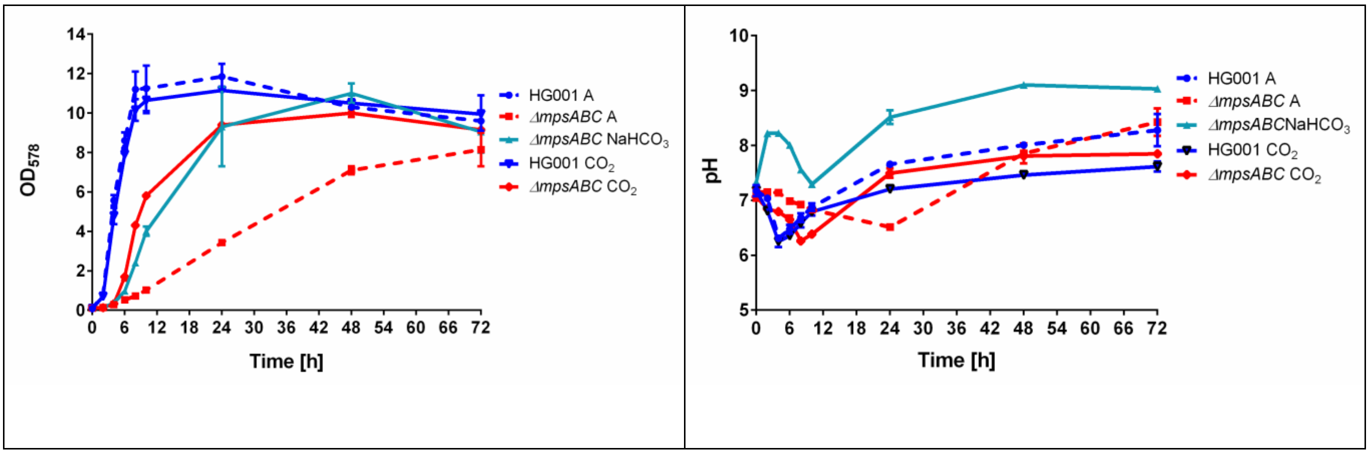

Supplementary Figure 8: Growth (left) and extracellular pH (right) of wild type HG001 and Δ*mpsABC* in atmospheric conditions (A) and 5% CO_2 and 20 mM $NaHCO_3$.

Each point shown in the graph represents the mean value ± standard error mean (SEM) from two independent experiments.

Supplementary References

1. Merlin C, Masters M, McAteer S, Coulson A. Why is carbonic anhydrase essential to *Escherichia coli*? *J Bacteriol* **185**, 6415-6424 (2003).

2. Herbert S*, et al.* Repair of global regulators in *Staphylococcus aureus* 8325 and comparative analysis with other clinical isolates. *Infect Immun* **78**, 2877-2889 (2010).

3. Diep BA*, et al.* Complete genome sequence of USA300, an epidemic clone of community-acquired meticillin-resistant *Staphylococcus aureus*. *Lancet* **367**, 731-739 (2006).

4. Baba T*, et al.* Genome and virulence determinants of high virulence community-acquired MRSA. *Lancet* **359**, 1819-1827 (2002).
